# Supplementary material for: Design of a randomised controlled trial: does indirect calorimetry energy information influence weight loss in obesity?
Source: BMJ Open. 2021 Mar 24;11(3):e044519. doi: 10.1136/bmjopen-2020-044519 (PMC7993246; doi:10.1136/bmjopen-2020-044519)
Supplement: Supplementary data [file bmjopen-2020-044519supp001.pdf]

RESTING METABOLIC PROFILE

Client Name: Participant No.
Date of Test: --/--/--

	Test Score	Normal Range	Optimal
Resting Metabolism (kcal)	2103	1564 - 1912	N/A
Resting Metabolism (kJ)	8805	6548 - 8005	N/A
Fat Burning (%)	90.4	48 - 83	80
Glucose Availability (%)	9.6	17 - 52	20
Efficiency (FEO2) (%)	15.62	16 - 17	< 15

Background

Energy production (metabolism) is vital to sustain every aspect of life and consequently is a key component of your health. For example, compromised energy production often results in an energy-related condition such as Obesity, Diabetes, Metabolic Syndrome, Chronic Fatigue, Insulin Resistance or a Sleep Disorder and may also be related to problems such hyperactivity, insomnia and an increased rate of aging.

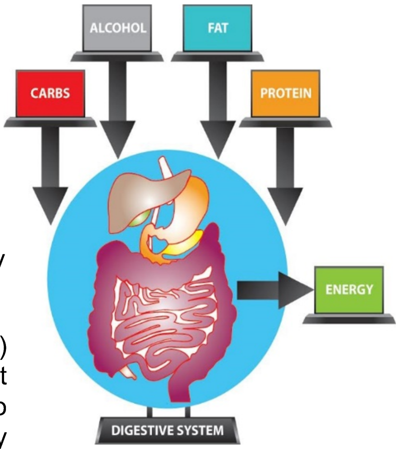

Therefore understanding and optimising your metabolism is key to improving your health, weight management and wellbeing.

As humans we obtain energy from the food and liquids (fuel) consumed. The digestion of these nutrients and the subsequent absorption makes it possible for the mitochondria in cells to transform the potential chemical energy of food into the energy we use for life.

This energy is normally produced from a mixture of glucose (from carbohydrates) and fat. At rest and light exercise the body should produce around 80% of the required energy from fat stores. Under high intensity exercise the body gradually switches from burning fats to glucose. The ratio of these two fuels is highly individual and is determined primarily by the amount of glucose available, but also hormone function, fitness, muscle mass and general health.

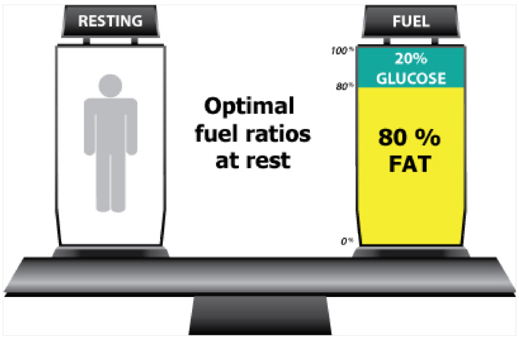

We have used a gold standard research technology called Indirect Calorimetry to measure your energy production. The device called ECAL allows our practitioners to accurately measure your daily energy requirement at rest, which nutrient source the energy is being produced from (fat and/or carbohydrate) and the efficiency with which your cells use oxygen.

Conventionally these energy requirements would be assessed using population based equations. However these are widely accepted as inaccurate because they cannot take into account individual variations in body composition, hormone function and physical activity levels. This can often lead to frustration for both client and practitioner.

Resting Energy Report of Participant

All of your test results are displayed in a table above, and where relevant normal or optimal values are also shown. However your metabolic profile is completely unique and therefore comparisons with these values are for general information only. Energy testing should be repeated over time and particularly following dietary or exercise intervention. This way you can measure the effectiveness of any strategy designed to improve your weight, energy and general health.

An explanation of each test score is provided and may include specific recommendations to optimize your energy production if necessary. Please consult with your health practitioner for appropriate diet, exercise or clinical intervention advice

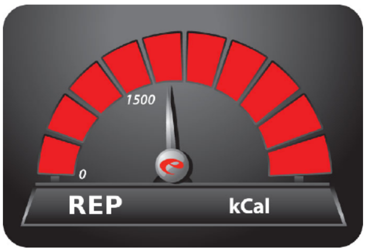

Resting Metabolism (REP/RMR) - This is the number of calories that would be expended over a 24 hour period if a person remained at rest/light activity. It usually represents around 65 - 80% of a person's total energy needs and is influenced by various factors including body composition, surface area, efficiency, age and hormones. ECAL accurately measures RMR and compares this against estimated values based on age, height, weight and sex.

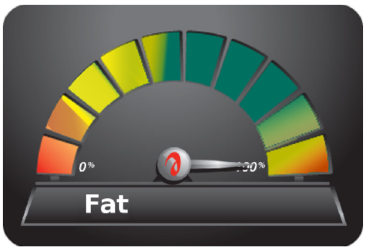

Fat Burning (%) - This score indicates the percentage of energy being generated from fat stores. The human body is designed to generate around 80% of total energy from fat stores at rest. This is particularly important for weight loss - to lose weight fat must be burnt. Factors that affect fat utilisation include diet, hormone function and physiological stress.

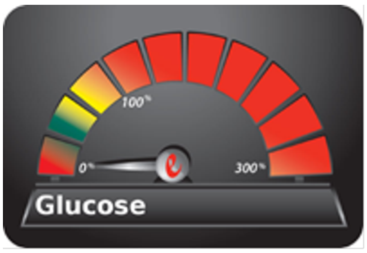

Glucose Availability (%) - This score indicates the amount of glucose (carbohydrate) currently available to generate energy. A surplus of glucose can result in elevated blood sugar levels, the storage of glucose and the conversion of glucose excess to fat, through a process known as lipogenesis.

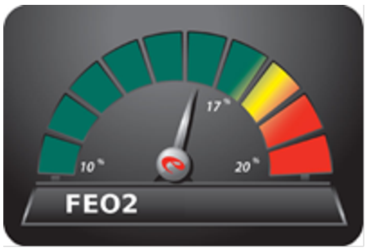

Efficiency (FEO2) - Our mitochondria (the part of the cell responsible for energy production) requires oxygen to convert the food we consume into useable energy. Healthier cells are more efficient and can therefore utilise more oxygen from the atmosphere - a lower value indicates more efficient mitochondria.

Recommendations to the Participant

RESTING Energy Expenditure

ECAL can accurately measure your REE and compare this against standard predictive equations.

High REE

A higher than expected resting energy level was recorded during the test.
Whilst high resting energy production generally results in better weight control it may be symptomatic of poor health. For example, high energy levels may cause symptoms such as hyperactivity, anxiety, insomnia, fatigue, low body weight and a difficulty maintaining muscle mass. The physiological reasons for recording a higher than normal resting energy level may include; (i) High mitochondrial function (FEO2 < 15%) (ii) Poor mitochondrial function (FEO2 > 17%) (iii) Fast breathing rate (RR > 15) (iv) Deep breathing (Ex Vol > 1.0L/breath)

RESPIRATORY QUOTIENT (RQ)

Normal RQ

A normal mean RQ score was recorded during the resting energy test.
It is common to read a normal RQ score and assume a state of good health, however the RQ score is calculated from two respiratory parameters; volume of expired carbon dioxide and the volume of oxygen consumed. It is therefore important to check that these two variables are also within normal range.

Are you having difficulty losing weight?

While RQ was measured as normal, weight control is dependent on other factors such as total calorie intake, insulin and the type of nutrients consumed.
It is very difficult to lose weight if one or all of the following conditions exist;
1. If the calorie intake is higher than the calorie expenditure.
2. If insulin is being released at rest.

Recommendations

Recommendations for 1:
a. Request the client accurately records their food and fluid intake over 5 - 7 consecutive days.
b. Calculate the client's average calorie intake per day and compare to their resting energy level. c. If the client's resting energy level is lower than their calorie intake, advise the client to increase their daily calorie expenditure by participating in moderate exercise for a minimum of 30 minutes per day and reduce calorie intake through reduced portion size.
Recommendations for 2:
a. Consider pathology testing of fasting insulin levels to confirm hyperinsulinaemia. b. Advise a reduction in the daily glucose intake.
c. Use the Glucose Calculator in the "Tools" section of ECHealth to calculate the client's glucose capacity and then prescribe 50 - 75% of the calculated liver capacity (grams) of glucose per day. Apply glucose restriction for 5 - 7 days.
d. Advise the client to participate in 20 - 30 minutes of moderate to intense exercise daily (as appropriate) to assist in lowering the high glucose level.
e. Repeat the resting energy test within 5 - 7 days to determine the effect of glucose restriction. f. If improved at the second test develop an eating plan based on 20% of the client's total daily energy expenditure (TEE = resting energy + energy expended through activity) to calculate the amount of glucose required per day.
g. If no change advice the client to undertake pathology testing of the sex hormones and cortisol in the morning around 8am to exclude a hormonal imbalance.
