# Supplementary material for: Design of a randomised controlled trial: does indirect calorimetry energy information influence weight loss in obesity?
Source: BMJ Open. 2021 Mar 24;11(3):e044519. doi: 10.1136/bmjopen-2020-044519 (PMC7993246; doi:10.1136/bmjopen-2020-044519)
Supplement: Author's manuscript [file bmjopen-2020-044519.draft_revisions.pdf]

BMJ Open is committed to open peer review. As part of this commitment we make the peer review history of every article we publish publicly available.

When an article is published we post the peer reviewers' comments and the authors' responses online. We also post the versions of the paper that were used during peer review. These are the versions that the peer review comments apply to.

The versions of the paper that follow are the versions that were submitted during the peer review process. They are not the versions of record or the final published versions. They should not be cited or distributed as the published version of this manuscript.

BMJ Open is an open access journal and the full, final, typeset and author-corrected version of record of the manuscript is available on our site with no access controls, subscription charges or pay-per-view fees (http://bmjopen.bmj.com).

If you have any questions on BMJ Open's open peer review process please email info.bmjopen@bmj.com

BMJ Open

Design of a Randomised Controlled Trial: Does Indirect Calorimetry Energy Information Influence Weight Loss in Obesity?

Journal:	*BMJ Open*
Manuscript ID	bmjopen-2020-044519
Article Type:	Protocol
Date Submitted by the Author:	05-Sep-2020
Complete List of Authors:	Lim, Jonathan; University of Liverpool Institute of Ageing and Chronic Disease, Department of Cardiovascular and Metabolic Medicine<br>Alam, Uazman; University of Liverpool Institute of Ageing and Chronic Disease, Department of Cardiovascular and Metabolic Medicine<br>Cuthbertson, Daniel; University of Liverpool Institute of Ageing and Chronic Disease, Department of Cardiovascular and Metabolic Medicine<br>Wilding, John; University of Liverpool Institute of Ageing and Chronic Disease, Department of Cardiovascular and Metabolic Medicine
Keywords:	Respiratory physiology < THORACIC MEDICINE, Diabetes & endocrinology < INTERNAL MEDICINE, Lipid disorders < DIABETES & ENDOCRINOLOGY

SCHOLARONE™
Manuscripts

I, the Submitting Author has the right to grant and does grant on behalf of all authors of the Work (as defined in the below author licence), an exclusive licence and/or a non-exclusive licence for contributions from authors who are: i) UK Crown employees; ii) where BMJ has agreed a CC-BY licence shall apply, and/or iii) in accordance with the terms applicable for US Federal Government officers or employees acting as part of their official duties; on a worldwide, perpetual, irrevocable, royalty-free basis to BMJ Publishing Group Ltd ("BMJ") its licensees and where the relevant Journal is co-owned by BMJ to the co-owners of the Journal, to publish the Work in this journal and any other BMJ products and to exploit all rights, as set out in our licence.

The Submitting Author accepts and understands that any supply made under these terms is made by BMJ to the Submitting Author unless you are acting as an employee on behalf of your employer or a postgraduate student of an affiliated institution which is paying any applicable article publishing charge ("APC") for Open Access articles. Where the Submitting Author wishes to make the Work available on an Open Access basis (and intends to pay the relevant APC), the terms of reuse of such Open Access shall be governed by a Creative Commons licence – details of these licences and which Creative Commons licence will apply to this Work are set out in our licence referred to above.

Other than as permitted in any relevant BMJ Author's Self Archiving Policies, I confirm this Work has not been accepted for publication elsewhere, is not being considered for publication elsewhere and does not duplicate material already published. I confirm all authors consent to publication of this Work and authorise the granting of this licence.

For peer review only

Design of a Randomised Controlled Trial: Does Indirect Calorimetry Energy Information Influence Weight Loss in Obesity?

Jonathan ZM Lim[1,2], Uazman Alam[1,2,3], Daniel Cuthbertson[1,2], John PH Wilding[1,2]

1 Department of Cardiovascular and Metabolic Medicine, Institute of Life Course and Medical Sciences, University of Liverpool, UK.

2 Division of Diabetes & Endocrinology, University Hospital Aintree, Liverpool University Hospitals NHS Foundation Trust, Liverpool, UK.

3 Department of Diabetes, Endocrinology & Gastroenterology, Faculty of Biology, Medicine and Health, University of Manchester, UK.

Corresponding author:
Dr Jonathan Lim,
Department of Cardiovascular and Metabolic Medicine,
Institute of Life Course and Medical Sciences,
University of Liverpool,
Longmoor Lane,
Liverpool, UK.
L9 7AL
Tel: +44 151 529 5940
E: j.z.lim@liverpool.ac.uk;

Word Count: 3496 words (Excluding Tables and References)

For peer review only

Abstract

Introduction

Restriction of energy intake relative to energy expenditure (EE) produces an energy deficit that is disproportionate to decrease in fat-free mass (FFM) and resting energy expenditure (REE). Expectations of weight change does not usually match predicted weight change due to this disproportionate reduction in energy expenditure. Respiratory quotient (RQ) serves an early biomarker of fat-to-carbohydrate metabolism, with estimated energy balance from REE. Our aim is to determine the effectiveness of utilising EE information from indirect calorimeter (IC) in weight management.

Methods and Analysis

Assessor-blinded, parallel-group randomised controlled trial of 105 adults with obesity randomised in 1:1 ratio to receive either standard weight management care (SC) or EE information plus SC (INT) during a 24-week multicomponent weight management program. The primary outcome is difference in weight loss between INT and SC group at 24-weeks. Secondary outcomes include: change in RQ, REE, glycaemic variability, and appetite-relating gut hormones (GLP-1, GIP, PYY). Generalised linear mixed models (intention-to-treat) will assess outcomes for treatment (INT vs. SC), time (baseline, 24-week) and the treatment-by-time interaction. This will be the first study to evaluate impact of utilising measured REE and RQ on the lifestyle-based intensive intervention program.

Ethics and dissemination

This study has been approved by the Health Research Authority and the North West Research Ethics Committee (18/NW/0645) and is conducted in accordance with the Declaration of Helsinki and Good Clinical Practice (GCP).

Trial registration number

ClinicalTrials.gov –NCT03638895; UoL001379

For peer review only

Strengths and limitations of this study

- There is limited evidence on the relationship between REE and RQ with body weight in context of intensive lifestyle intervention for weight loss in adults with obesity and severe obesity.
- The study is a well-designed assessor-blinded, parallel-group randomised controlled trial to assess the impact of providing timely EE information from IC to promote behaviour modification for weight loss within a specialist weight management service (SWMS) in the UK.
- The study selection and randomisation process is structured and statistically powered based on previous data representative of our local SWMS population.
- There is a robust statistical analysis plan that will follow intention-to-treat principles and per protocol analysis.

For peer review only

Article Summary

What is already known about this subject?

The change in energy expenditure is not directly proportionate the change in body weight in obesity and severe obesity. There is limited evidence to evaluate the relationship between RQ and REE with change in body weight with intensive lifestyle intervention for weight loss in adults. Some evidence suggests RQ is a useful early biomarker to predict change in body weight. There is a disproportionate reduction in REE in response to actual vs. predicted weight loss. There is a lack of well-designed randomised-controlled studies which directly examined the impact of providing EE information i.e. RQ and REE from IC on body weight in the context of lifestyle intervention for weight loss in adults with obesity.

What is the key question?

Does providing indirect calorimeter energy expenditure information influence body weight in patients with obesity and severe obesity undergoing structured lifestyle intervention for weight loss?

How might this impact on clinical practice in the foreseeable future?

The ability to adapt and provide timely and effective EE information from IC to promote behaviour modification for weight loss could be a useful adjunct in SWMS.

Keywords

Energy Expenditure; Respiratory Quotient; Obesity; Indirect Calorimetry; Fat-Free Mass; Body Weight

Abbreviations

EE – Energy Expenditure

FEO2 – fraction of oxygen inspired

FECO2 – fraction of carbon dioxide expired

FFM – fat-free mass

IC – Indirect calorimeter

INT – Intervention Group

REE – Resting Energy Expenditure

RQ – Respiratory Quotient

SC – Standard Care Group

VO2 – volume of oxygen consumed

VCO2- volume of carbon dioxide consumed

For peer review only

Introduction

The prevalence of obesity has reached pandemic levels both worldwide and in the UK, having more than doubled globally over the last three decades (1). Reducing the obesity-related health burden as well as tackling the obesity pandemic is core to WHO 'Global Action Plan for Prevention and Control of Non-Communicable Diseases 2013-2020'(2). The obesogenic environment is a consequence of industrialisation of labour and food systems, leading to over-consumption of energy-dense, nutrient-poor foods combined with an increasingly sedentary lifestyle (3). Behaviour-centred weight loss through intensive lifestyle modifications has proven effective in the short-term in attaining modest but clinically significant weight loss (4, 5). However, excessive energy intake over prolonged period leads to inability to maintain the consistent negative energy deficit required to achieve weight loss. Individuals seeking weight loss encounter much difficulty due to Increased hunger and a disproportionate reduction in energy expenditure (EE) driven by neuro-hormonal adaptations (6). Consequently, maintenance of weight loss is difficult and weight regain common (4, 7).

Practical methods to better engage with people using information, self-directed education and promote effective weight loss are continually evolving amongst specialist weight management programmes. The ECAL indirect calorimeter (IC) (Metabolic Health Solutions Pty Ltd, Perth, Australia) is an open-circuit portable device that measures fractional volume exchange in oxygen consumed (VO2) and carbon dioxide expired (VCO2) within a small mixing chamber(8). The ECAL IC uses a proprietary mouth piece and nose clip. O2 measurements were calculated with a galvanic fuel cell oxygen sensor and CO2 measurements were obtained using a novel LED non-dispersive IR sensor(9). VO2 and VCO2 gas exchange measurements were derived from physiological methods based on Weir's equation(10). Data generated from breath-by-breath exchange during a steady

For peer review only - http://bmjopen.bmj.com/site/about/guidelines.xhtml

volumetric flow whilst in an energy-balanced resting state, generates the resting energy expenditure (REE) and respiratory quotient (RQ). ECAL IC has been validated against other IC including the GEM and DeltaTrac validating its reliability (8). Comparing between devices, coefficient of variance was 4 (±5.3)% on the Deltatrac, 4.9 (±4.5)% on the GEM and 11.2 (±12.1)% on the ECAL(8). Deltatrac was previously considered the reference standard after validation studies demonstrated low bias and good precision in comparison to Douglas bag method, however the Deltatrac is now discontinued from commercial sale(11). The RQ is the index of VCO2 against VO2 which is expressed as a ratio. Typically, metabolism consisting solely of fat or lipids generates an RQ of 0.70 - 0.75. The approximate RQ of mixed metabolism of fat and carbohydrate is 0.80. Likewise, protein metabolism generates approximate RQ of 0.80. Metabolism consisting solely of carbohydrate produces RQ of 1.0.

Longitudinal prospective studies reported the association between low RQ (<0.75) with weight loss and weight loss maintenance (12-15). Numerous epidemiological and experimental studies further support this correlation that a lower RQ predicts weight loss (13, 16-18). Goris et al. reported post-absorptive RQ was strongly related to change in body mass (r=0.57; P=0.0001), but not BMI, fat mass or fat-free mass (19). Conversely, adjusting for variables of gender, ethnicity, familial predisposition, those with higher RQ were associated with weight gain or struggled to maintain the loss (12, 16). In the Baltimore Longitudinal study in Aging, Seidell et al. reported that high fasting RQ was a weak but significant predictor of weight gain(16). In previously overweight and obese subjects, high RQ predicted an increase in body fat mass independent of energy balance, circulating insulin and insulin sensitivity (20, 21). Marra et al. reported baseline RQ in non-obese women was a significant predictor of body weight (P<0.05) after a 6-year follow-up period (21). Hainer *et al.* reported that obese subjects on very low calorie diet with high RQ regained weight at the 2-year follow up(22).

For peer review only

Based on the current evidence in literature, RQ serves as a valuable indicator of substrate oxidation during an energy-balanced state.

Behavioural weight loss programs classically involve decreased calorie intake, increased energy expenditure and use behavioural strategies such as goal setting and self-monitoring. To our knowledge, this is the first study that has been specifically designed to investigate the utilisation of energy expenditure information (RQ & REE) from the ECAL IC on the change in body weight. We postulate use of individualised energy expenditure information generated from the portable ECAL indirect calorimeter will result in greater weight change behaviour modification.

This paper describes the protocol and statistical analysis plan for the Energy Expenditure from ECAL Indirect Calorimeter in a Multicomponent Weight Management Service (NCT03638895). This is a RCT incorporating the use of EE information from the ECAL IC versus standard care (SC) in participants with obesity and severe obesity attending a secondary care based specialist weight management service (SWMS).

Aims and Objectives

The aim of the ECAL study is to determine the effectiveness of utilising energy expenditure (EE) information from ECAL indirect calorimetry to influence the outcome of weight loss in obese non-diabetic individuals receiving dietary restriction, exercise and behaviour modification therapy.

The secondary objectives are to compare the health outcomes in terms of: (i) association of weight with measured RQ; (ii) association of weight change with measured REE; (iii) change

For peer review only

in glycaemic variability; (iv) change in appetite-regulating gut hormones (glucagon-like peptide 1 (GLP-1), gastric inhibitory polypeptide (GIP), peptide YY (PYY).

Methods

Study Design

Study design is a 24-week parallel-group randomised controlled trial. The study protocol version 1.2 (Date: 24-JUL-2018) has been approved by the Health Research Authority (HRA) and the North West Research Ethics Committee (REC 18/NW/0645) and is prospectively registered with Clinical Trials Registry (NCT03638895). The design, conduct and reporting of this study has been approved and sponsored by the University of Liverpool / Liverpool Joint Research Office.

Trial Oversight and Governance

The study sponsor is the University of Liverpool and the study is managed and overseen by the University of Liverpool / Liverpool Joint Research Office. The study is registered at the http://www.clinicaltrials.gov (NCT03638895) before the enrolment of the first participant and monitored by the University of Liverpool / Liverpool Joint Research Office. Safety of the participants will be monitored by the University of Liverpool / Liverpool Joint Research Office. Decision for any interim data analysis and stopping guidelines will be assessed and validated through the University of Liverpool/ Liverpool Joint Research Office.

Participants and recruitment

Recruitment of participants will take place over 24 months from February 2019 to February 2021 in Liverpool University Hospitals NHS Foundation Trust, Liverpool, United Kingdom.

For peer review only

Participants will be recruited through current referrals to Aintree LOSS community-based weight management service, face-to-face clinic encounters, group-based education sessions, electronic database of participants attending the Tier 3 specialist weight management service, and notices in the hospital. Participants with BMIs ≥ 30 kg/m^2 will be identified from referral lists and weight management clinics. Participants will receive a study letter which briefly explains study aims and advises that researchers will be in contact within 2 weeks to provide further details of the study. If participants do not respond to study advertising within 6 months of commencement of recruitment, there will be a further broadening of recruitment sites to include local weight management services subject to prior ethical approval. All individuals identified will be given a patient information leaflet and required to provide written informed consent prior to enrolment. People interested in participating will be met by researchers at the study site to obtain consent and complete the baseline questionnaires.

Patient and Public Involvement

Patients were involved in the design and conduct of this research. During the feasibility stage, priority of the research question, choice of outcome measures, and methods of recruitment were informed by discussions with patients through two focus group sessions. Members of the Tier 3 specialist weight management service at Liverpool University Hospitals NHS Trust also identified this research as being a priority area for clinicians and patients undergoing weight management intervention. Once the trial has been published, participants will be informed of the results through the local trust website and will be sent details of the results in a study newsletter suitable for a layperson audience.

Eligibility

For peer review only

To determine eligibility, the participants must fulfil all the eligibility criteria (Table 1). Criteria were designed to ensure that participants were able to safely engage with the weight management programme.

The following exclusion criteria apply (Table 1):

1) participants taking medications interfering with weight loss (including orlistat, phentermine, buprenorphine/naltrexone, sibutramine, GLP-1 agonists, corticosteroids)

2) history of weight loss surgery including bariatric surgery (gastric band, roux-en-Y gastric bypass, duodenal switch operation)

3) type 1 or type 2 diabetes mellitus (defined as HbA1c ≥48 mmol/mol)

4) uncontrolled thyroid-dysfunction (TSH <0.4 or >6.0 mIU/L)

The principal investigator and sub-investigators will determine eligibility of each participant before entering the study.

Blinding

Both participants and researchers will be masked to the ECAL results in the SC arm. It will not be possible to 'mask' researchers or participants to group allocation. Those responsible for analysis will be masked to group allocation.

Control Group (Standard Care) (SC)

Participants in the SC group will receive multidisciplinary weight management intervention including dietary intervention, recommendations of physical activity and behaviour modification advise. Dietary restrictions will be estimated based upon the Harris-Benedict equation for total daily energy requirements, with a recommended 500 kcal/day energy deficit. Typically, suggested meal plans range from approximately 1,200 to 2,500 kcal per day. Dietetic adivse will be based upon the Association of UK dieticians (BDA)(23)

For peer review only

recommendation on daily intake of carbohydrates (40-60%), protein (20-30%), and fat (20-30%). Participants will be encouraged not to have prolonged episodes of fasting, but to maintain up to three regular portioned balanced meals/day, while limiting snacks and reinforcing portion control. Dietician will provide the participant with a food diary and offer them self-monitoring tools to monitor diet using MyFitnessPal™ Mobile App (www.myfitnesspal.com) for at least two days per week. Up to 150 minutes of physical activity/week will be recommended. Physical activity will be adjusted based on comorbid conditions including osteoarthritis and muscular disorders. Participants will be encouraged to formulate personalised dietary restriction and achievable activity goals each week during each study visit. In order to make a comparison of indirect measurements of REE and RQ between groups, the participants receiving SC will also undergo indirect calorimetry measurement, but without receiving the EE information. For full details on assessments during study visits, refer to Table 2.

Intervention Group (INT)

The intervention group participants will receive EE information generated from ECAL IC in addition to the lifestyle-based interventions described in the SC group. EE information components will encompass the REE and RQ delivered in the form of a layperson client report containing summary of recommendations to help improve understanding about metabolic health (example of client report can be found in supplementary material in Appendix). During the study visit, these results will serve as a resource for both clinicians and participants, as an adjunctive tool to deliver key messages to improve participants' understanding of body metabolism and EE. The compiled client report will include change in REE, RQ and fat vs. carbohydrate oxidation based on their measured anthropometric data at each visit. This resource will serve as a reference tool for the clinician and participant when

For peer review only

making informed choices about behaviour modification in order to achieve their target weight loss. For full details on assessments during study visits, refer to Table 2.

Study Outcomes

Primary Outcome

The primary outcome is the difference in change of weight between participants in the INT group (EE information plus SC) versus the SC group at baseline and 24-weeks after randomisation.

Secondary Measurements and Outcomes

Anthropometrics, weight change, body composition (fat mass, fat-free mass), obesity-associated comorbidities, changes in REE, RQ and substrate (fat and glucose oxidation) based on indirect calorimetry, will be measured and determined throughout the study. These are listed in the Supplementary Material, Appendix 1.

Indirect Calorimetry Protocol

Prior to each IC measurement, all participants will receive detailed preparatory oral and printed information. Participants will be asked to abstain from food, alcohol, and any calorie-containing beverages for at least 8 h and refrain from vigorous physical activity. Participants will arrive between 10 – 12 hours after an overnight fast before undergoing the IC test. Participants will be asked to confirm their fasting duration and will be reminded to follow preparatory procedures prior to next visit. The ECAL indirect calorimeter (Metabolic Health Solutions Pty Ltd, Australia) used will be calibrated using the 5% carbon dioxide to calibrate gas flow, as per manufacturer specifications. Bio impedance analysis (TANITA Body Impedance Analyser) determining fat mass and lean body mass will be undertaken.

For peer review only

Anthropometric data will be entered into the EC Health software linked up to the ECAL IC. Participant will be lying comfortably in a supine position and elevated at a 45-degrees angle. After 15 minutes, a nose clip will be applied over the nostrils and participant will be instructed to breathe through a single-use mouthpiece, ensuring a tight seal over it. The test instructor will observe the volumetric flow and ensure that a steady volume and rate of breathing is maintained for between 8 to 10 minutes. A successful test will be defined as a steady-state achieved with a minimum of 5 consecutive minutes with less than 10% coefficient of variation in FEO_2 and $FECO_2$(24). The instructor will stop the indirect calorimetry measurement when this steady-state has been achieved. Should any interruptions occur or failing to achieve a steady-state, a repeat testing will be performed.

Client Report

A client report will be generated using the EC Health software when the IC measurement is complete. A series of questions related to their time-specific metabolism will be completed and a client report generated for participant and clinician in the intervention group only. An example of client report can be found in Supplementary Material, Appendix.

Meal Test

All participants will be offered the option of participating in a meal test based upon their calculated total daily energy requirement. After an overnight fast of at least 8 hours, participants will be offered Ensure Plus milkshake which consists of a 220mls of milkshake style oral nutritional supplement with energy content of 1.5kcal/ml. Ensure Plus consist of 17% calories from fat, 17% from carbohydrate and 26% from protein.

For peer review only

Blood samples will be taken to determine baseline concentrations of plasma glucose, insulin and appetite-related gut hormones including glucagon-like peptide-1 (GLP-1), gastric inhibitory polypeptide (GIP) and peptide YY (PYY). Once completed, the meal test will consist of 35% of the participant's calculated 24-hour energy requirement in the form of the Ensure Plus milkshake. Blood samples will then be taken at 15, 30, 60, 90 and 120 minutes after the meal ingestion.

For peer review only

Glycaemic Variability

We will investigate the secondary impact of weight loss on glycaemic variability in a subset of obese individuals with prediabetes and compare the changes in mean amplitude of glycaemic excursion (MACE) using Continuous Glucose Monitoring (CGM). Only the participants with prediabetes, defined as glycated haemoglobin HbA1c of 42 – 47 mmol/mol will be recruited for this sub-group study.

STUDY POPULATION

The sample size calculation is based upon retrospective dataset from the SWMS, with a derived standard deviation of 4kg. Thus, 42 participants in each group will give our study 80% power to detect a difference in weight change between groups of 3kg at the 5% significance level using a two sided test. A between-group difference of 3kg was chosen as this is outside the range of normal weight fluctuation and is sufficient to sustain clinically meaningful weight loss (5). Accounting for attrition rate of 20%, the total target recruitment is 105 participants.

STATISTICAL METHODS

Statistical analysis will be performed using IBM SPSS Statistics version 20 or later. All variables will be checked for plausibility and missing values. Outcome data will be presented

as mean (SD) for continuous variables and counts (percentages) for categorical variables. Differences between groups at randomisation and characteristics of completers versus will be tested using independent *t*-tests for between group analyses and paired t-test for within group analyses. A non-parametric counterpart will be used if data are not normally distributed. Chi-squared (χ^2) tests will be utilised for categorical variables.

PRIMARY ANALYSIS PLAN

Linear mixed models will be used to assess weight and other secondary outcomes for the impact of intervention (EE information plus SC vs. SC alone), time (treated as categorical with levels at baseline, 3 and 6 months), fat-free mass, resting energy expenditure and treatment-by-time interaction, with these terms forming the base model. This will ensure that the outcomes for participants who drop out of the program or "lost to follow-up" are retained in the analyses, in line with an intention-to-treat approach and per protocol analysis. Age, body mass index, obesity-related comorbidities (King's Obesity Staging Criteria) and quality of life outcomes and glycaemic variability will be examined to determine whether they contribute significantly to the models. If a covariate is significant, a term will be added to the model to adjust for the effects and two-way interactions with time and treatment will also be examined. If these interactions are also significant they will be similarly adjusted for in the model.

SECONDARY ANALYSES PLAN – MEDIATION ANALYSIS

Hypothesised psychological and behavioural mediators of the weight loss intervention effect will be examined in SPSS. Regression coefficients will be used to reflect (i) the effect of the intervention on the hypothesised mediator (ii) the association between the hypothesised mediator and the outcome, controlling for treatment condition, and (iii) the total, direct and

For peer review only

indirect intervention effects. Macro generates bias-corrected 95% confidence intervals around the indirect effect. Sample size required for the RCT will provide adequate power for this analysis to detect medium sized mediation effects.

RANDOMISATION

Participants will be randomised at an individual level by an independent statistician without contact with participants during the trial. Using a computer-based random number-producing algorithm, allocation sequence within will be generated to allow equal ratio of 1:1 to either the intervention or control group. Randomisation for each stratum will be performed within balanced blocks into BMI category (30-39.9 kg/m^2 and ≥ 40.0kg/m^2) and sex (male and female participants). Complete separation will be achieved between the statistician who generated the randomisation sequence and those involved in assessing participants and performing data entry.

ALLOCATION

A research assistant who will not be involved in the enrolment, assessment or allocation of participants will pack and sequence pre-packed envelopes with the group allocation. When study participants complete all baseline assessments, a study investigator/ sub-investigator will review laboratory reports and summary of health records from their primary care practitioner and assess concomitant medications. Only after an investigator/ sub-investigator confirms eligibility to proceed, will the envelope be opened and details of the particular study group will be revealed to the participant. If the participant is randomised into the intervention group, layperson client printouts and summary will then be provided with their program resources.

For peer review only

DISCUSSION

The study goal is to assess the impact of utilising REE and RQ from IC as an adjunctive management for weight loss. The role of utilising EE information to influence behaviour has yet to be evaluated within a real-world pragmatic RCT. Further, the results will help advance existing evidence about the relationship between RQ, REE and change in body weight.

Several experimental studies evaluating change in REE as a response to overfeeding (i.e. 40-70% increase from baseline energy requirements) suggested that short-term (2-8 weeks) overfeeding produces over-compensatory increase in REE, such that the increase in REE is greater than predicted in relation to changes in body composition and weight (25-29). Such compensatory increase in energy intake lead to weight increases, of which 55-67% constitutes fat mass gain (25, 26, 30, 31). Conversely, underfeeding studies reveal that in short-term, intentional weight loss leads to decrease in EE beyond predicted values(32-34). Decrease in REE is disproportionate to weight measured per kilogram against FFM after weight loss. The regression analysis of change in REE against the change in FFM reveals that decline in REE tended to be greater than accounted for by loss of FFM. Such over-compensatory metabolic changes act to oppose further weight change (35).

Variability in substrate oxidation between individuals may be an inherent variability that contributes towards weight regain and fat storage. Variability of macronutrient composition intake between participants may influence the measured RQ and influence the results associated with fat vs. carbohydrate oxidation(36). Several studies have suggested macronutrient alterations could also influence appetite-regulation (37, 38). Further, individual differences in circulating insulin levels or sensitivity may confound the reported associations

For peer review only

between RQ, REE and fat mass(15, 39, 40). We have taken this into account and have therefore excluded patients with diabetes mellitus or metabolic disorders.

Indirect calorimetry use has been validated through several detailed experimental in vitro and in vivo studies, demonstrating accuracy of measuring EE when compared to the mass spectrometry and Douglas Bag method(41). Given that previous studies on other commercial metabolic monitors showed variations in gas exchange rates(42), leading to secondary metabolic results variability, participants will be invited to strictly adhere to the protocol requirements and all measurements performed under controlled and steady-state experimental setting.

The strength of the proposed study is the RCT design with extensive detail on a statistical analysis plan that will follow intention-to-treat principles and per protocol analysis. In addition to measuring weight and other physiological parameters, the trial will include assessment of detailed secondary outcome measures.

Competing interests

J.P.H.W. reports personal fees, grants, and consultancy fees paid to his institution from AstraZeneca, Novo Nordisk, and Takeda, personal fees and consultancy fees paid to his institution from Boehringer Ingelheim, Janssen, Napp, Mundipharma, and Sanofi, and consultancy fees paid to his institution from Lilly, Rhythm Pharmaceuticals and Wilmington Healthcare outside of the submitted work.

J.Z.M.L., U.A., D.J.C. has no competing interests to declare.

Author contributions

For peer review only - http://bmjopen.bmj.com/site/about/guidelines.xhtml

The study chief investigators J.Z.M.L., U.A., D.J.C., and J.P.H.W were responsible for creating the research question, design of the study, obtaining ethical approval, the acquisition of funding and oversight to study implementation. All authors were responsible for the drafting of this manuscript.

Acknowledgements & Funding

The ECAL RCT was funded by the University of Liverpool (Grant: UoL001379). The ECAL indirect calorimeter was provided and maintained by the manufacturer Metabolic Health Solutions, Australia. The funding bodies did not have any input into the design of the study, the collection or analysis of data, the preparation of this manuscript or the decision to submit this manuscript for publication.

For peer review only

Table 1: Inclusion and exclusion criteria from protocol

Inclusion criteria

- Man or woman, 18 to 70 years of age
- BMI ≥30 kg/ m^2 to ≤ 60 kg/m^2 at screening visit
- Stable weight (change of < 5% within 12 weeks before screening based on medical history)
- Subjects are in the investigators opinion, well-motivated, capable, and willing to learn how to undergo indirect calorimetry testing, as required for study
- Willing and able to adhere to the prohibitions and restrictions specified within this protocol

Exclusion criteria

- Taking weight loss medication within 12 weeks prior to randomisation
- Previous or planned bariatric surgery
- History of type 1, type 2 diabetes mellitus, diabetic ketoacidosis, or diabetes secondary to pancreatitis
- Has a HbA1c of ≥ 6.5% (or ≥ 48 mmol/mol)
- History of obesity with a known secondary cause (e.g. Cushing's disease / syndrome)
- Oral corticosteroid use (except in the short term use of a 7-10 day course)
- Ongoing, inadequately controlled thyroid disorder defined as thyroid-stimulating hormone >6mIU/litre or <0.4 mIU/litre
- History of malignancy within 3 years before screening (or diagnosis of malignancy within this period)
- eGFR ≤30ml/min/1.73m^2 on serum testing
- Alanine aminotransferase level is >2.0 times the upper limit of normal or total bilirubin is >1.5 times the upper limit of normal at screening
- Other major illness likely to preclude participation in the trial
- History of glucagonoma
- A myocardial infarction, unstable angina, revascularization procedure (stent or bypass graft surgery) or cerebrovascular accident within 12 weeks before screening
- Heart Failure NYHA Class III-IV
- End-stage Chronic Obstructive Pulmonary Disease (COPD)

BMI, Body mass index; HbA1c, glycosylated haemoglobin; eGFR, estimated glomerular filtration rate; NYHA, New York Heart Association.

For peer review only

Table 2: Assessments and procedures during study visits.

**Procedure** Visit window(days)	**SCR** ±7	**W0**	**W1** ±3	**W2** ±3	**W4** ±3	**W8** ±3	**W12** ±3	**W16** ±3	**W20** ±3	**W24** ±3
**Visit**	**1**	**2**	**3**	**4**	**5**	**6**	**7**	**8**	**9**	**10**
Informed consent	X									
Inclusion exclusion criteria	X	X								
Randomisation criteria and randomisation		X								
Medical History	X									
Physical examination	X									
Indirect calorimetry		X	X	X	X	X	X	X	X	X
Full blood count	X									
Renal profile	X									
Lipid profile		X								X
Liver function test	X									X
Thyroid function	X									
GLP1, GIP, PYY		X								X
Fasting glucose		X								X
HbA1c	X									X
Urine pregnancy test	X									
Height	X									
Weight	X	X	X	X	X	X	X	X	X	X
Waist, hip, thigh circumference		X		X	X	X	X	X	X	X
FFM, FM		X		X	X	X	X	X	X	X
Blood pressure	X	X			X	X	X	X	X	X
Diet counselling		X			X	X	X	X	X	X
Food Diary		X	X	X	X	X	X	X	X	X
Compliance check			X	X	X	X	X	X	X	X
Physical Activity check		X			X	X	X	X	X	X
Meal Test		X								X
CGM		X			X		X			X
Concomitant medication check	X	X	X	X	X	X	X	X	X	X
AE reporting		X			X	X	X	X	X	X
KOSC	X									X
ESS	X									X
STOP-BANG	X									X
IWQoL-Lite		X								X
EuroQoL-5D-5L		X								X

For peer review only

Acceptability survey	X	X

SCR – Screening visit; W0 – Week 0; W1 – Week 1; W2 – Week 2; W4 – Week 4; W8 – Week 8; W12 – Week 12; W16 – Week 16; W20 – Week 20; W24 – Week 24
FFM – Fat-free mass; FM – Fat mass; CGM – Continuous Glucose Monitor; AE – Adverse Events Reporting; KOSC, King's obesity staging score; ESS, Epworth sleep score; STOP-BANG – questionnaire for sleep apnoea assessment; IWQoL-Lite- Questionnaire for quality of life; EuroQoL-5D-5L – Questionnaire for Quality of life

For peer review only

References

1. Ng M, Fleming T, Robinson M, Thomson B, Graetz N, Margono C, et al. Global, regional, and national prevalence of overweight and obesity in children and adults during 1980–2013: a systematic analysis for the Global Burden of Disease Study 2013. The Lancet. 2014;384(9945):766-81.
2. World Health Organisation. Obesity and Overweight 2016 [Available from: https://www.who.int/news-room/fact-sheets/detail/obesity-and-overweight.

3. Hruby A, Hu FB. The Epidemiology of Obesity: A Big Picture. Pharmacoeconomics. 2015;33(7):673-89.
4. Wing RR, Phelan S. Long-term weight loss maintenance. Am J Clin Nutr. 82. United States2005. p. 222S-5S.
5. Wing RR, Lang W, Wadden TA, Safford M, Knowler WC, Bertoni AG, et al. Benefits of Modest Weight Loss in Improving Cardiovascular Risk Factors in Overweight and Obese Individuals With Type 2 Diabetes. Diabetes Care. 2011;34(7):1481-6.
6. Sumithran P, Prendergast LA, Delbridge E, Purcell K, Shulkes A, Kriketos A, et al. Long-term persistence of hormonal adaptations to weight loss. N Engl J Med. 2011;365(17):1597-604.
7. Leibel RL, Rosenbaum M, Hirsch J. Changes in energy expenditure resulting from altered body weight. N Engl J Med. 1995;332(10):621-8.
8. Kennedy S, Ryan L, Fraser A, Clegg ME. Comparison of the GEM and the ECAL indirect calorimeters against the Deltatrac for measures of RMR and diet-induced thermogenesis. J Nutr Sci. 2014;3:e52.
9. Macdonald-Clarke CJ, O'Hagan D, Amirabdollahian F. Measurement of resting energy expenditure using novel, portable indirect calorimetry device ECAL compared with conventional measurements by QUARK RMR and prediction equation formulae. Proceedings of the Nutrition Society. 2016;75(OCE3):E101.
10. Weir JB. New methods for calculating metabolic rate with special reference to protein metabolism. J Physiol. 1949;109(1-2):1-9.
11. Tissot S, Delafosse B, Bertrand O, Bouffard Y, Viale JP, Annat G. Clinical validation of the Deltatrac monitoring system in mechanically ventilated patients. Intensive Care Med. 1995;21(2):149-53.
12. Valtueña S, Salas-Salvadó J, Lorda PG. The respiratory quotient as a prognostic factor in weight-loss rebound. International Journal of Obesity. 1997;21(9):811-7.
13. Zurlo F, Lillioja S, Esposito-Del Puente A, Nyomba BL, Raz I, Saad MF, et al. Low ratio of fat to carbohydrate oxidation as predictor of weight gain: study of 24-h RQ. Am J Physiol. 1990;259(5 Pt 1):E650-7.
14. Toubro S, Sørensen TIA, Hindsberger C, Christensen NJ, Astrup A. Twenty-Four-Hour Respiratory Quotient: The Role of Diet and Familial Resemblance1. The Journal of Clinical Endocrinology & Metabolism. 1998;83(8):2758-64.
15. Galgani J, Ravussin E. Energy metabolism, fuel selection and body weight regulation. Int J Obes (Lond). 2008;32 Suppl 7(Suppl 7):S109-19.
16. Seidell JC, Muller DC, Sorkin JD, Andres R. Fasting respiratory exchange ratio and resting metabolic rate as predictors of weight gain: the Baltimore Longitudinal Study on Aging. Int J Obes Relat Metab Disord. 1992;16(9):667-74.
17. Filozof CM, Murúa C, Sanchez MP, Brailovsky C, Perman M, Gonzalez CD, et al. Low plasma leptin concentration and low rates of fat oxidation in weight-stable post-obese subjects. Obes Res. 2000;8(3):205-10.
18. Chaput JP, Pérusse L, Després JP, Tremblay A, Bouchard C. Findings from the Quebec Family Study on the Etiology of Obesity: Genetics and Environmental Highlights. Curr Obes Rep. 2014;3(1):54-66.
19. Goris AH, Westerterp KR. Postabsorptive respiratory quotient and food quotient-an analysis in lean and obese men and women. Eur J Clin Nutr. 2000;54(7):546-50.
20. Ellis AC, Hyatt TC, Hunter GR, Gower BA. Respiratory quotient predicts fat mass gain in premenopausal women. Obesity (Silver Spring). 2010;18(12):2255-9.

For peer review only

21. Marra M, Scalfi L, Contaldo F, Pasanisi F. Fasting Respiratory Quotient as a Predictor of Long-Term Weight Changes in Non-Obese Women. Annals of Nutrition and Metabolism. 2004;48(3):189-92.
22. Hainer V, Kunesová M, Parízková J, Stich V, Mikulová R, Slabá S. Respiratory quotient in obesity: its association with an ability to retain weight loss and with parental obesity. Sb Lek. 2000;101(1):99-104.
23. BDA Obesity Specialist Group. Dietetic Obesity Management Interventions in Adults: Evidence Review & Clinical Application.2018:[1-102 pp.].
24. Popp CJ, Butler M, Curran M, Illiano P, Sevick MA, St-Jules DE. Evaluating steady-state resting energy expenditure using indirect calorimetry in adults with overweight and obesity. Clinical Nutrition. 2019.
25. Ravussin E, Schutz Y, Acheson KJ, Dusmet M, Bourquin L, Jéquier E. Short-term, mixed-diet overfeeding in man: no evidence for "luxuskonsumption". Am J Physiol. 1985;249(5 Pt 1):E470-7.
26. Norgan NG, Durnin JV. The effect of 6 weeks of overfeeding on the body weight, body composition, and energy metabolism of young men. Am J Clin Nutr. 1980;33(5):978-88.
27. Dériaz O, Fournier G, Tremblay A, Després JP, Bouchard C. Lean-body-mass composition and resting energy expenditure before and after long-term overfeeding. Am J Clin Nutr. 1992;56(5):840-7.
28. Bray GA, Smith SR, de Jonge L, Xie H, Rood J, Martin CK, et al. Effect of dietary protein content on weight gain, energy expenditure, and body composition during overeating: a randomized controlled trial. Jama. 2012;307(1):47-55.
29. Leaf A, Antonio J. The Effects of Overfeeding on Body Composition: The Role of Macronutrient Composition - A Narrative Review. Int J Exerc Sci. 2017;10(8):1275-96.
30. Johannsen DL, Tchoukalova Y, Tam CS, Covington JD, Xie W, Schwarz JM, et al. Effect of 8 weeks of overfeeding on ectopic fat deposition and insulin sensitivity: testing the "adipose tissue expandability" hypothesis. Diabetes Care. 2014;37(10):2789-97.
31. Roberts SB, Young VR, Fuss P, Fiatarone MA, Richard B, Rasmussen H, et al. Energy expenditure and subsequent nutrient intakes in overfed young men. Am J Physiol. 1990;259(3 Pt 2):R461-9.
32. Heshka S, Yang MU, Wang J, Burt P, Pi-Sunyer FX. Weight loss and change in resting metabolic rate. Am J Clin Nutr. 1990;52(6):981-6.
33. Van Gaal LF, Vansant GA, De Leeuw IH. Factors determining energy expenditure during very-low-calorie diets. Am J Clin Nutr. 1992;56(1 Suppl):224s-9s.
34. Buscemi S, Verga S, Caimi G, Cerasola G. Low relative resting metabolic rate and body weight gain in adult Caucasian Italians. Int J Obes (Lond). 2005;29(3):287-91.
35. Weyer C, Pratley RE, Salbe AD, Bogardus C, Ravussin E, Tataranni PA. Energy expenditure, fat oxidation, and body weight regulation: a study of metabolic adaptation to long-term weight change. J Clin Endocrinol Metab. 2000;85(3):1087-94.
36. Schutz Y. Abnormalities of fuel utilization as predisposing to the development of obesity in humans. Obes Res. 1995;3 Suppl 2:173s-8s.
37. Kozimor A, Chang H, Cooper JA. Effects of dietary fatty acid composition from a high fat meal on satiety. Appetite. 2013;69:39-45.
38. Blundell JE, Stubbs RJ, Golding C, Croden F, Alam R, Whybrow S, et al. Resistance and susceptibility to weight gain: individual variability in response to a high-fat diet. Physiol Behav. 2005;86(5):614-22.

For peer review only

39. Thalacker-Mercer AE, Ingram KH, Guo F, Ilkayeva O, Newgard CB, Garvey WT. BMI, RQ, diabetes, and sex affect the relationships between amino acids and clamp measures of insulin action in humans. Diabetes. 2014;63(2):791-800.
40. Camastra S, Bonora E, Del Prato S, Rett K, Weck M, Ferrannini E. Effect of obesity and insulin resistance on resting and glucose-induced thermogenesis in man. EGIR (European Group for the Study of Insulin Resistance). Int J Obes Relat Metab Disord. 1999;23(12):1307-13.
41. Graf S, Karsegard VL, Viatte V, Heidegger CP, Fleury Y, Pichard C, et al. Evaluation of three indirect calorimetry devices in mechanically ventilated patients: which device compares best with the Deltatrac II(®)? A prospective observational study. Clin Nutr. 2015;34(1):60-5.
42. Cooper JA, Watras AC, O'Brien MJ, Luke A, Dobratz JR, Earthman CP, et al. Assessing validity and reliability of resting metabolic rate in six gas analysis systems. J Am Diet Assoc. 2009;109(1):128-32.

For peer review only

RESTING METABOLIC PROFILE

Client Name: Participant No.
Date of Test: --/--/--

	Test Score	Normal Range	Optimal
Resting Metabolism (kcal)	2103	1564 - 1912	N/A
Resting Metabolism (kJ)	8805	6548 - 8005	N/A
Fat Burning (%)	90.4	48 - 83	80
Glucose Availability (%)	9.6	17 - 52	20
Efficiency (FEO2) (%)	15.62	16 - 17	< 15

Background

Energy production (metabolism) is vital to sustain every aspect of life and consequently is a key component of your health. For example, compromised energy production often results in an energy-related condition such as Obesity, Diabetes, Metabolic Syndrome, Chronic Fatigue, Insulin Resistance or a Sleep Disorder and may also be related to problems such hyperactivity, insomnia and an increased rate of aging.

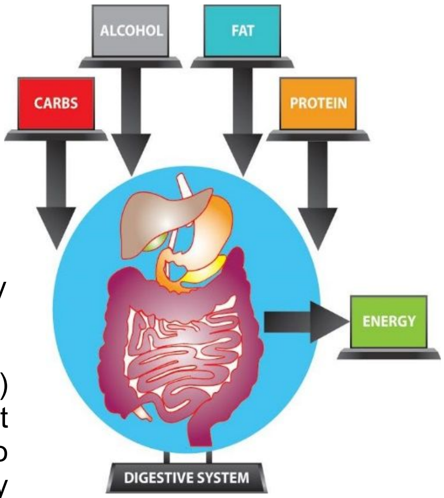

Therefore understanding and optimising your metabolism is key to improving your health, weight management and wellbeing.

As humans we obtain energy from the food and liquids (fuel) consumed. The digestion of these nutrients and the subsequent absorption makes it possible for the mitochondria in cells to transform the potential chemical energy of food into the energy we use for life.

This energy is normally produced from a mixture of glucose (from carbohydrates) and fat. At rest and light exercise the body should produce around 80% of the required energy from fat stores. Under high intensity exercise the body gradually switches from burning fats to glucose. The ratio of these two fuels is highly individual and is determined primarily by the amount of glucose available, but also hormone function, fitness, muscle mass and general health.

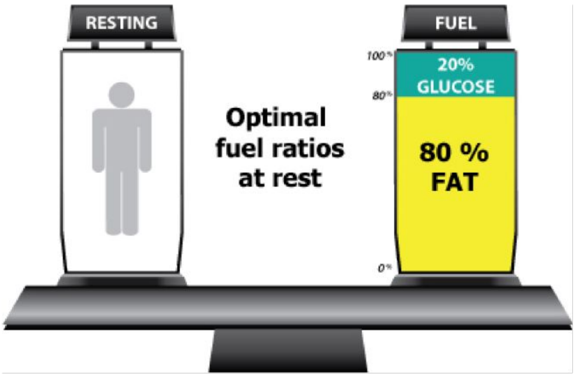

We have used a gold standard research technology called Indirect Calorimetry to measure your energy production. The device called ECAL allows our practitioners to accurately measure your daily energy requirement at rest, which nutrient source the energy is being produced from (fat and/or carbohydrate) and the efficiency with which your cells use oxygen.

Conventionally these energy requirements would be assessed using population based equations. However these are widely accepted as inaccurate because they cannot take into account individual variations in body composition, hormone function and physical activity levels. This can often lead to frustration for both client and practitioner.

For peer review only

Resting Energy Report of Participant

All of your test results are displayed in a table above, and where relevant normal or optimal values are also shown. However your metabolic profile is completely unique and therefore comparisons with these values are for general information only. Energy testing should be repeated over time and particularly following dietary or exercise intervention. This way you can measure the effectiveness of any strategy designed to improve your weight, energy and general health.

An explanation of each test score is provided and may include specific recommendations to optimize your energy production if necessary. Please consult with your health practitioner for appropriate diet, exercise or clinical intervention advice

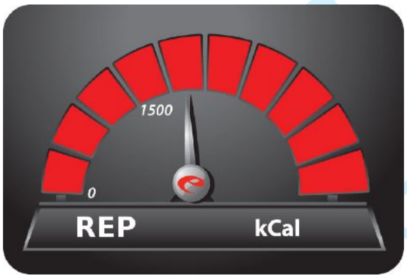

Resting Metabolism (REP/RMR) - This is the number of calories that would be expended over a 24 hour period if a person remained at rest/light activity. It usually represents around 65 - 80% of a person's total energy needs and is influenced by various factors including body composition, surface area, efficiency, age and hormones. ECAL accurately measures RMR and compares this against estimated values based on age, height, weight and sex.

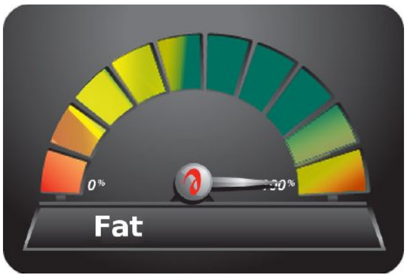

Fat Burning (%) - This score indicates the percentage of energy being generated from fat stores. The human body is designed to generate around 80% of total energy from fat stores at rest. This is particularly important for weight loss - to lose weight fat must be burnt. Factors that affect fat utilisation include diet, hormone function and physiological stress.

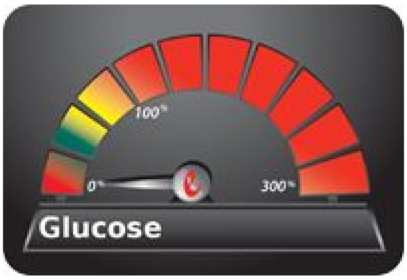

Glucose Availability (%) - This score indicates the amount of glucose (carbohydrate) currently available to generate energy. A surplus of glucose can result in elevated blood sugar levels, the storage of glucose and the conversion of glucose excess to fat, through a process known as lipogenesis.

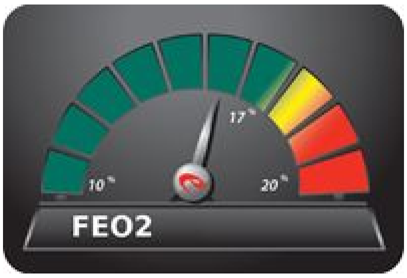

Efficiency (FEO2) - Our mitochondria (the part of the cell responsible for energy production) requires oxygen to convert the food we consume into useable energy. Healthier cells are more efficient and can therefore utilise more oxygen from the atmosphere - a lower value indicates more efficient mitochondria.

Recommendations to the Participant

RESTING Energy Expenditure

ECAL can accurately measure your REE and compare this against standard predictive equations.

High REE

A higher than expected resting energy level was recorded during the test.

Whilst high resting energy production generally results in better weight control it may be symptomatic of poor health. For example, high energy levels may cause symptoms such as hyperactivity, anxiety, insomnia, fatigue, low body weight and a difficulty maintaining muscle mass. The physiological reasons for recording a higher than normal resting energy level may include; (i) High mitochondrial function (FEO2 < 15%) (ii) Poor mitochondrial function (FEO2 > 17%) (iii) Fast breathing rate (RR > 15) (iv) Deep breathing (Ex Vol > 1.0L/breath)

RESPIRATORY QUOTIENT (RQ)

Normal RQ

A normal mean RQ score was recorded during the resting energy test.

It is common to read a normal RQ score and assume a state of good health, however the RQ score is calculated from two respiratory parameters; volume of expired carbon dioxide and the volume of oxygen consumed. It is therefore important to check that these two variables are also within normal range.

Are you having difficulty losing weight?

While RQ was measured as normal, weight control is dependent on other factors such as total calorie intake, insulin and the type of nutrients consumed.
It is very difficult to lose weight if one or all of the following conditions exist;

1. If the calorie intake is higher than the calorie expenditure.
2. If insulin is being released at rest.

Recommendations

Recommendations for 1:

a. Request the client accurately records their food and fluid intake over 5 - 7 consecutive days.

b. Calculate the client's average calorie intake per day and compare to their resting energy level. c. If the client's resting energy level is lower than their calorie intake, advise the client to increase their daily calorie expenditure by participating in moderate exercise for a minimum of 30 minutes per day and reduce calorie intake through reduced portion size.

Recommendations for 2:

For peer review only

a. Consider pathology testing of fasting insulin levels to confirm hyperinsulinaemia. b. Advise a reduction in the daily glucose intake.
c. Use the Glucose Calculator in the "Tools" section of ECHealth to calculate the client's glucose capacity and then prescribe 50 - 75% of the calculated liver capacity (grams) of glucose per day. Apply glucose restriction for 5 - 7 days.
d. Advise the client to participate in 20 - 30 minutes of moderate to intense exercise daily (as appropriate) to assist in lowering the high glucose level.
e. Repeat the resting energy test within 5 - 7 days to determine the effect of glucose restriction. f. If improved at the second test develop an eating plan based on 20% of the client's total daily energy expenditure (TEE = resting energy + energy expended through activity) to calculate the
amount of glucose required per day.
g. If no change advice the client to undertake pathology testing of the sex hormones and cortisol in the morning around 8am to exclude a hormonal imbalance.

For peer review only

Reporting checklist for protocol of a clinical trial.

Based on the SPIRIT guidelines.

Instructions to authors

Complete this checklist by entering the page numbers from your manuscript where readers will find each of the items listed below.

Your article may not currently address all the items on the checklist. Please modify your text to include the missing information. If you are certain that an item does not apply, please write "n/a" and provide a short explanation.

Upload your completed checklist as an extra file when you submit to a journal.

In your methods section, say that you used the SPIRITreporting guidelines, and cite them as:

Chan A-W, Tetzlaff JM, Altman DG, Laupacis A, Gøtzsche PC, Krleža-Jerić K, Hróbjartsson A, Mann H, Dickersin K, Berlin J, Doré C, Parulekar W, Summerskill W, Groves T, Schulz K, Sox H, Rockhold FW, Rennie D, Moher D. SPIRIT 2013 Statement: Defining standard protocol items for clinical trials. Ann Intern Med. 2013;158(3):200-207

		Reporting Item	Page Number
**Administrative information**			
Title	#1	Descriptive title identifying the study design, population, interventions, and, if applicable, trial acronym	1
Trial registration	#2a	Trial identifier and registry name. If not yet registered, name of intended registry	8
Trial registration: data set	#2b	All items from the World Health Organization Trial Registration Data Set	8
Protocol version	#3	Date and version identifier	8
Funding	#4	Sources and types of financial, material, and other support	8
Roles and responsibilities: contributorship	#5a	Names, affiliations, and roles of protocol contributors	1

For peer review only

Roles and responsibilities: sponsor contact information	#5b	Name and contact information for the trial sponsor	1
Roles and responsibilities: sponsor and funder	#5c	Role of study sponsor and funders, if any, in study design; collection, management, analysis, and interpretation of data; writing of the report; and the decision to submit the report for publication, including whether they will have ultimate authority over any of these activities	8
Roles and responsibilities: committees	#5d	Composition, roles, and responsibilities of the coordinating centre, steering committee, endpoint adjudication committee, data management team, and other individuals or groups overseeing the trial, if applicable (see Item 21a for data monitoring committee)	8
**Introduction**			
Background and rationale	#6a	Description of research question and justification for undertaking the trial, including summary of relevant studies (published and unpublished) examining benefits and harms for each intervention	5-7
Background and rationale: choice of comparators	#6b	Explanation for choice of comparators	5-7
Objectives	#7	Specific objectives or hypotheses	7
Trial design	#8	Description of trial design including type of trial (eg, parallel group, crossover, factorial, single group), allocation ratio, and framework (eg, superiority, equivalence, non-inferiority, exploratory)	7-8
**Methods: Participants, interventions, and outcomes**			
Study setting	#9	Description of study settings (eg, community clinic, academic hospital) and list of countries where data will be collected. Reference to where list of study sites can be obtained	8-9
Eligibility criteria	#10	Inclusion and exclusion criteria for participants. If applicable, eligibility criteria for study centres and individuals who will	9

For peer review only

		perform the interventions (eg, surgeons, psychotherapists)	
Interventions: description	#11a	Interventions for each group with sufficient detail to allow replication, including how and when they will be administered	9-10
Interventions: modifications	#11b	Criteria for discontinuing or modifying allocated interventions for a given trial participant (eg, drug dose change in response to harms, participant request, or improving / worsening disease)	9-10
Interventions: adherance	#11c	Strategies to improve adherence to intervention protocols, and any procedures for monitoring adherence (eg, drug tablet return; laboratory tests)	9-10
Interventions: concomitant care	#11d	Relevant concomitant care and interventions that are permitted or prohibited during the trial	9-10
Outcomes	#12	Primary, secondary, and other outcomes, including the specific measurement variable (eg, systolic blood pressure), analysis metric (eg, change from baseline, final value, time to event), method of aggregation (eg, median, proportion), and time point for each outcome. Explanation of the clinical relevance of chosen efficacy and harm outcomes is strongly recommended	11
Participant timeline	#13	Time schedule of enrolment, interventions (including any run-ins and washouts), assessments, and visits for participants. A schematic diagram is highly recommended (see Figure)	11
Sample size	#14	Estimated number of participants needed to achieve study objectives and how it was determined, including clinical and statistical assumptions supporting any sample size calculations	13
Recruitment	#15	Strategies for achieving adequate participant enrolment to reach target sample size	8
**Methods: Assignment of interventions (for controlled trials)**			
Allocation: sequence generation	#16a	Method of generating the allocation sequence (eg, computer-generated random numbers), and list of any factors for stratification. To reduce predictability of a random sequence, details of any planned restriction (eg, blocking) should be provided in a separate document that is unavailable to those who enrol participants or assign interventions	15

For peer review only

Allocation concealment mechanism	#16b	Mechanism of implementing the allocation sequence (eg, central telephone; sequentially numbered, opaque, sealed envelopes), describing any steps to conceal the sequence until interventions are assigned	15
Allocation: implementation	#16c	Who will generate the allocation sequence, who will enrol participants, and who will assign participants to interventions	15
Blinding (masking)	#17a	Who will be blinded after assignment to interventions (eg, trial participants, care providers, outcome assessors, data analysts), and how	15
Blinding (masking): emergency unblinding	#17b	If blinded, circumstances under which unblinding is permissible, and procedure for revealing a participant's allocated intervention during the trial	15
**Methods: Data collection, management, and analysis**			
Data collection plan	#18a	Plans for assessment and collection of outcome, baseline, and other trial data, including any related processes to promote data quality (eg, duplicate measurements, training of assessors) and a description of study instruments (eg, questionnaires, laboratory tests) along with their reliability and validity, if known. Reference to where data collection forms can be found, if not in the protocol	14
Data collection plan: retention	#18b	Plans to promote participant retention and complete follow-up, including list of any outcome data to be collected for participants who discontinue or deviate from intervention protocols	14
Data management	#19	Plans for data entry, coding, security, and storage, including any related processes to promote data quality (eg, double data entry; range checks for data values). Reference to where details of data management procedures can be found, if not in the protocol	14
Statistics: outcomes	#20a	Statistical methods for analysing primary and secondary outcomes. Reference to where other details of the statistical analysis plan can be found, if not in the protocol	14
Statistics: additional	#20b	Methods for any additional analyses (eg, subgroup and adjusted	14

For peer review only

analyses		analyses)	
Statistics: analysis population and missing data	#20c	Definition of analysis population relating to protocol non-adherence (eg, as randomised analysis), and any statistical methods to handle missing data (eg, multiple imputation)	14
**Methods: Monitoring**			
Data monitoring: formal committee	#21a	Composition of data monitoring committee (DMC); summary of its role and reporting structure; statement of whether it is independent from the sponsor and competing interests; and reference to where further details about its charter can be found, if not in the protocol. Alternatively, an explanation of why a DMC is not needed	17
Data monitoring: interim analysis	#21b	Description of any interim analyses and stopping guidelines, including who will have access to these interim results and make the final decision to terminate the trial	17
Harms	#22	Plans for collecting, assessing, reporting, and managing solicited and spontaneously reported adverse events and other unintended effects of trial interventions or trial conduct	17
Auditing	#23	Frequency and procedures for auditing trial conduct, if any, and whether the process will be independent from investigators and the sponsor	17
**Ethics and dissemination**			
Research ethics approval	#24	Plans for seeking research ethics committee / institutional review board (REC / IRB) approval	8
Protocol amendments	#25	Plans for communicating important protocol modifications (eg, changes to eligibility criteria, outcomes, analyses) to relevant parties (eg, investigators, REC / IRBs, trial participants, trial registries, journals, regulators)	8
Consent or assent	#26a	Who will obtain informed consent or assent from potential trial participants or authorised surrogates, and how (see Item 32)	8
Consent or assent: ancillary studies	#26b	Additional consent provisions for collection and use of participant data and biological specimens in ancillary studies, if applicable	8

For peer review only

Confidentiality	#27	How personal information about potential and enrolled participants will be collected, shared, and maintained in order to protect confidentiality before, during, and after the trial	8
Declaration of interests	#28	Financial and other competing interests for principal investigators for the overall trial and each study site	18
Data access	#29	Statement of who will have access to the final trial dataset, and disclosure of contractual agreements that limit such access for investigators	17
Ancillary and post trial care	#30	Provisions, if any, for ancillary and post-trial care, and for compensation to those who suffer harm from trial participation	17
Dissemination policy: trial results	#31a	Plans for investigators and sponsor to communicate trial results to participants, healthcare professionals, the public, and other relevant groups (eg, via publication, reporting in results databases, or other data sharing arrangements), including any publication restrictions	17
Dissemination policy: authorship	#31b	Authorship eligibility guidelines and any intended use of professional writers	17
Dissemination policy: reproducible research	#31c	Plans, if any, for granting public access to the full protocol, participant-level dataset, and statistical code	17
**Appendices**			
Informed consent materials	#32	Model consent form and other related documentation given to participants and authorised surrogates	17
Biological specimens	#33	Plans for collection, laboratory evaluation, and storage of biological specimens for genetic or molecular analysis in the current trial and for future use in ancillary studies, if applicable	17

None The SPIRIT checklist is distributed under the terms of the Creative Commons Attribution License CC-BY-ND 3.0. This checklist can be completed online using https://www.goodreports.org/, a tool made by the EQUATOR Network in collaboration with Penelope.ai

For peer review only

BMJ Open

Design of a Randomised Controlled Trial: Does Indirect Calorimetry Energy Information Influence Weight Loss in Obesity?

Journal:	*BMJ Open*
Manuscript ID	bmjopen-2020-044519.R1
Article Type:	Protocol
Date Submitted by the Author:	08-Feb-2021
Complete List of Authors:	Lim, Jonathan; University of Liverpool Institute of Ageing and Chronic Disease, Department of Cardiovascular and Metabolic Medicine; University Hospital Aintree, Department of Diabetes & Endocrinology<br>Alam, Uazman; University of Liverpool Institute of Ageing and Chronic Disease, Department of Cardiovascular and Metabolic Medicine; University Hospital Aintree, Department of Diabetes & Endocrinology<br>Cuthbertson, Daniel; University of Liverpool Institute of Ageing and Chronic Disease, Department of Cardiovascular and Metabolic Medicine; University Hospital Aintree, Department of Diabetes & Endocrinology<br>Wilding, John; University of Liverpool Institute of Ageing and Chronic Disease, Department of Cardiovascular and Metabolic Medicine; University Hospital Aintree, Department of Diabetes & Endocrinology
**Primary Subject Heading**:	Nutrition and metabolism
Secondary Subject Heading:	Diabetes and endocrinology
Keywords:	NUTRITION & DIETETICS, DIABETES & ENDOCRINOLOGY, Respiratory physiology < THORACIC MEDICINE

SCHOLARONE™
Manuscripts

I, the Submitting Author has the right to grant and does grant on behalf of all authors of the Work (as defined in the below author licence), an exclusive licence and/or a non-exclusive licence for contributions from authors who are: i) UK Crown employees; ii) where BMJ has agreed a CC-BY licence shall apply, and/or iii) in accordance with the terms applicable for US Federal Government officers or employees acting as part of their official duties; on a worldwide, perpetual, irrevocable, royalty-free basis to BMJ Publishing Group Ltd ("BMJ") its licensees and where the relevant Journal is co-owned by BMJ to the co-owners of the Journal, to publish the Work in this journal and any other BMJ products and to exploit all rights, as set out in our licence.

The Submitting Author accepts and understands that any supply made under these terms is made by BMJ to the Submitting Author unless you are acting as an employee on behalf of your employer or a postgraduate student of an affiliated institution which is paying any applicable article publishing charge ("APC") for Open Access articles. Where the Submitting Author wishes to make the Work available on an Open Access basis (and intends to pay the relevant APC), the terms of reuse of such Open Access shall be governed by a Creative Commons licence – details of these licences and which Creative Commons licence will apply to this Work are set out in our licence referred to above.

Other than as permitted in any relevant BMJ Author's Self Archiving Policies, I confirm this Work has not been accepted for publication elsewhere, is not being considered for publication elsewhere and does not duplicate material already published. I confirm all authors consent to publication of this Work and authorise the granting of this licence.

For peer review only

Design of a Randomised Controlled Trial: Does Indirect Calorimetry Energy Information Influence Weight Loss in Obesity?

Jonathan ZM Lim[1,2], Uazman Alam[1,2], Daniel Cuthbertson[1,2], John PH Wilding[1,2]

1 Department of Cardiovascular and Metabolic Medicine, Institute of Life Course and Medical Sciences, University of Liverpool, UK.

2 Division of Diabetes & Endocrinology, University Hospital Aintree, Liverpool University Hospitals NHS Foundation Trust, Liverpool, UK.

Corresponding author:
Dr Jonathan Lim,
Department of Cardiovascular and Metabolic Medicine,
Institute of Life Course and Medical Sciences,
University of Liverpool,
Longmoor Lane,
Liverpool, UK.
L9 7AL
Tel: +44 151 529 5940
E: j.z.lim@liverpool.ac.uk;

Word Count: 5842 words (excluding tables and references)

For peer review only

For peer review only - http://bmjopen.bmj.com/site/about/guidelines.xhtml

Abstract

Introduction

Respiratory quotient (RQ) provides an indication of the relative balance of carbohydrate and fat oxidation. RQ could serve as an early biomarker of negative energy balance during weight loss. Restriction of energy intake relative to total daily energy requirements produces a negative energy balance which can lead to a fall in RQ, accompanied by a decrease in resting energy expenditure (REE). However, the net change in body weight does not usually match predicted weight change due to intra-individual metabolic adaptations. Our aim is to determine the effectiveness of utilising energy expenditure (EE) information from indirect calorimetry during weight loss intervention.

Methods and Analysis

We will undertake an assessor-blinded, parallel-group randomised controlled trial of 105 adults with obesity randomised in 1:1 ratio to receive either standard weight management care (SC) or EE information plus SC (INT) during a 24-week multicomponent weight management program. The primary outcome is difference in weight loss between INT and SC group at 24-weeks. Secondary outcomes include: change in RQ, REE, glycaemic variability, and appetite-relating gut hormones (GLP-1, GIP, PYY). Generalised linear mixed models (intention-to-treat) will assess outcomes for treatment (INT vs. SC), time (baseline, 24-week) and the treatment-by-time interaction. This will be the first study to evaluate impact of utilising measured REE and RQ on the lifestyle-based intensive intervention program.

Ethics and dissemination

Ethics approval was obtained from the Health Research Authority and the North West Research Ethics Committee (18/NW/0645). Results from this trial will be disseminated through publication in peer-reviewed journals, national and international presentations.

Trial registration number

For peer review only - http://bmjopen.bmj.com/site/about/guidelines.xhtml

ClinicalTrials.gov –NCT03638895; UoL001379

Strengths and limitations of this study

- This will be the first randomised controlled trial to evaluate whether providing energy expenditure (EE) information during interactions between dietician and participants can influence weight loss during a weight loss intervention in people with obesity.
- Exploratory information on a broad range of outcomes will be evaluated including but not limited to change in body weight, body composition, resting energy expenditure and substrate oxidation which will inform the design of future weight management trials.
- The results of study will advance our current understanding of the impact of utilising EE information to inform or predict body weight change and substrate oxidation in people with obesity.
- A limitation of the study design is that the indirect calorimetry only provides information about the EE based on the time-constraints of those settings (fasted and resting state) only which may not reflect day-to-day variations in EE due to influence of energy intake and physical activity.
- Whilst the ECAL indirect calorimetry is a portable device and more convenient to use than the ventilated hood systems, it has relatively wide limits of agreement and degree of variance between repeated measures of RMR, RQ and substrate oxidation and thus may be less informative than more precise measures of EE.

Keywords

Energy Expenditure; Respiratory Quotient; Obesity; Indirect Calorimetry; Fat-Free Mass; Body Weight

For peer review only - http://bmjopen.bmj.com/site/about/guidelines.xhtml

Abbreviations

EE – Energy Expenditure

FEO_2 – fraction of oxygen inspired

$FECO_2$ – fraction of carbon dioxide expired

FFM – fat-free mass

IC – Indirect calorimeter

INT – Intervention Group

REE – Resting Energy Expenditure

RQ – Respiratory Quotient

SC – Standard Care Group

SWMS – Specialist weight management service

VO_2 – volume of oxygen consumed

VCO_2- volume of carbon dioxide consumed

Introduction

The prevalence of obesity has reached pandemic levels both worldwide and in the UK, having more than doubled globally over the last three decades (1). Reducing the obesity-related health burden as well as tackling the obesity pandemic is core to WHO 'Global Action Plan for Prevention and Control of Non-Communicable Diseases 2013-2020' (2). The obesogenic environment is a consequence of industrialisation of labour and food systems, leading to over-consumption of energy-dense, nutrient-poor foods combined with an increasingly sedentary lifestyle (3). Behaviour-centred weight loss through intensive lifestyle modifications has proven effective in the short-term in attaining modest but clinically significant weight loss (4, 5). However, excessive energy intake over prolonged period leads to inability to maintain the consistent negative energy deficit required to achieve weight loss. Individuals seeking

For peer review only - http://bmjopen.bmj.com/site/about/guidelines.xhtml

weight loss encounter much difficulty due to increased hunger and a disproportionate reduction in energy expenditure (EE) driven by neuro-hormonal adaptations (6). Consequently, maintenance of weight loss is difficult and weight regain common (4, 7).

Practical methods to better engage with people using information, self-directed education and promote effective weight loss are continually evolving amongst specialist weight management programmes. The ECAL indirect calorimeter (IC) (Metabolic Health Solutions Pty Ltd, Perth, Australia) is an open-circuit portable device that measures fractional volume exchange in oxygen consumed (VO_2) and carbon dioxide expired (VCO_2) within a small mixing chamber(8). The ECAL IC uses a proprietary mouth piece and nose clip. O_2 measurements were calculated with a galvanic fuel cell oxygen sensor and CO_2 measurements were obtained using a novel LED non-dispersive IR sensor. VO_2 and VCO_2 gas exchange measurements were derived from physiological methods based on Weir's equation(9). Data generated from breath-by-breath exchange during a steady volumetric flow whilst in an energy-balanced resting state, generates the resting energy expenditure (REE) and respiratory quotient (RQ). ECAL IC has been compared against other IC including the GEM and DeltaTrac. Kennedy et al. (8) reported that measures of VO2, RMR, RQ, carbohydrate oxidation, and fat oxidation showed greatest variation on the ECAL IC as compared against Deltatrac (as the standard reference device). The mean difference in RMR measures collected on ECAL and Deltatrac showed wide limits of agreement (lower 95% limit of agreement was -2562 kJ/d and upper 95% limit of agreement was 3480 kJ/d). A greater proportional bias was observed between measured RMR of ECAL against Deltatrac suggesting that at higher RMR the difference between the two devices was greater but was acceptable for repeat RMR measures between individuals. In terms of repeatability of measures of ECAL IC (intra-machine variability), Kennedy et al. reported that no significant

For peer review only - http://bmjopen.bmj.com/site/about/guidelines.xhtml

differences were found between repeated measures of VCO2, RMR, RQ and substrate oxidation measures. However, greatest bias occurred within the ECAL with a mean difference of 475 ± 1083 kJ/d and wide limits of agreement (-2641 and 1691 kJ/d). Comparing between devices, coefficient of variance was 4 (±5.3)% on the Deltatrac, 4.9 (±4.5)% on the GEM and 11.2 (±12.1)% on the ECAL (8). Deltatrac was previously considered the reference standard after validation studies demonstrated low bias and good precision in comparison to Douglas bag method, however the Deltatrac is now discontinued from commercial sale (10). The RQ is the index of VCO_2 against VO_2 which is expressed as a ratio. Typically, metabolism consisting solely of fat or lipids generates an RQ of 0.70 - 0.75. The approximate RQ of mixed metabolism of fat and carbohydrate is 0.80. Likewise, protein metabolism generates approximate RQ of 0.80. Metabolism consisting solely of carbohydrate produces RQ of 1.0.

Longitudinal prospective studies reported the association between low RQ (<0.75) with weight loss and weight loss maintenance (11-14). Numerous epidemiological and experimental studies further support this correlation that a lower RQ predicts weight loss (12, 15-17). Goris et al. reported post-absorptive RQ was strongly related to change in body mass (r=0.57; P=0.0001), but not BMI, fat mass or fat-free mass (18). Conversely, adjusting for variables of sex, ethnicity, familial predisposition, those with higher RQ were associated with weight gain or struggled to maintain the loss (11, 15). In the Baltimore Longitudinal study in Aging, Seidell et al. reported that high fasting RQ was a weak but significant predictor of weight gain (15). In previously overweight and obese subjects, high RQ predicted an increase in body fat mass independent of energy balance, circulating insulin and insulin sensitivity (19, 20). Marra et al. reported baseline RQ in non-obese women was a significant predictor of body weight (P<0.05) after a 6-year follow-up period (20). Hainer *et al.* reported that people with obesity on very low calorie diet with high RQ regained weight at the 2-year follow up

For peer review only - http://bmjopen.bmj.com/site/about/guidelines.xhtml

(21). Based on the current evidence in literature, RQ serves as a valuable indicator of substrate oxidation during an energy-balanced state. If substrate oxidation was examined in a positive energy balance state, i.e. energy intake > expenditure, typically carbohydrate oxidation increases and fat oxidation decreases (22). Conversely, if measured in a negative energy balance state i.e. energy intake < expenditure, carbohydrate oxidation decreases and fat oxidation increases (23). The predominant factors that influences the accuracy of RQ measure of substrate oxidation is determined by body composition, energy intake and EE (24).

Behavioural weight loss programs classically involve decreased calorie intake, increased EE and use behavioural strategies such as goal setting and self-monitoring. This is the first study that has been specifically designed to investigate the utilisation of energy expenditure information (REE and RQ) from the ECAL IC on the change in body weight. We postulate use of individualised EE information generated from the portable ECAL IC will result in greater weight change behaviour modification.

This paper describes the protocol and statistical analysis plan for the randomised controlled trial of EE from ECAL IC in a Multicomponent Weight Management Service (NCT03638895). This RCT incorporates the use of EE information from the ECAL IC versus standard care (SC) in participants with obesity and severe obesity attending a secondary care based specialist weight management service (SWMS).

OBJECTIVES

Primary objective

For peer review only - http://bmjopen.bmj.com/site/about/guidelines.xhtml

The primary aim of the study is to determine the effectiveness of providing EE information from ECAL IC to influence the outcome of weight loss in obese individuals without diabetes receiving dietary restriction, exercise and behaviour modification therapy.

Secondary objectives

The secondary objectives are to compare the health outcomes in terms of: (i) association of weight change with the change in measured RQ; (ii) association of weight change with change in measured REE; (iii) change in glycaemic variability; (iv) change in appetite-regulating gut hormones (glucagon-like peptide 1 (GLP-1), gastric inhibitory polypeptide (GIP), and peptide YY (PYY).

METHODS AND ANALYSIS

Study Design

Study design is a 24-week parallel-group randomised controlled trial. The study protocol version 1.3 (Date: 28/08/2020) has been approved by the Health Research Authority (HRA) and the North West Research Ethics Committee (REC 18/NW/0645) and is prospectively registered with Clinical Trials Registry (NCT03638895). The design, conduct and reporting of this study has been approved and sponsored by the University of Liverpool / Liverpool Joint Research Office.

Patient and Public Involvement

Patients were involved in the design and conduct of this research. During the feasibility stage, priority of the research question, choice of outcome measures, and methods of recruitment were informed by discussions with patients through two focus group sessions. Members of the Tier 3 specialist weight management service at Liverpool University Hospitals NHS Trust also identified this research as being a priority area for clinicians and patients undergoing

For peer review only

weight management intervention. Once the trial has been published, participants will be informed of the results through the local trust website and will be sent details of the results in a study newsletter suitable for a layperson audience.

Trial Oversight and Governance

The study sponsor is the University of Liverpool and the study is managed and overseen by the University of Liverpool / Liverpool Joint Research Office. The study is registered at the http://www.clinicaltrials.gov (NCT03638895) before the enrolment of the first participant and monitored by the University of Liverpool / Liverpool Joint Research Office. Safety of the participants will be monitored by the University of Liverpool / Liverpool Joint Research Office. Decision for any interim data analysis and stopping guidelines will be assessed and validated through the University of Liverpool/ Liverpool Joint Research Office.

Participants

Eligibility Criteria

To determine eligibility, the participants must fulfil all the eligibility criteria (Table 1). Criteria were designed to ensure that participants were able to safely engage with the weight management programme.

Recruitment

Recruitment of participants will take place from February 2019 to February 2022 in Liverpool University Hospitals NHS Foundation Trust, Liverpool, United Kingdom. Participants will be recruited through current referrals to Aintree LOSS community-based weight management service, face-to-face clinic encounters, group-based education sessions, electronic database of participants attending the Tier 3 specialist weight management service, and notices in the

For peer review only - http://bmjopen.bmj.com/site/about/guidelines.xhtml

hospital. Participants with BMIs ≥ 30 kg/m^2 will be identified from referral lists and weight management clinics. Participants will receive a study letter which briefly explains study aims and advises that researchers will be in contact within 2 weeks to provide further details of the study. If participants do not respond to study advertising within 6 months of commencement of recruitment, there will be a further broadening of recruitment sites to include local weight management services subject to prior ethical approval. All individuals identified will be given a patient information leaflet and required to provide written informed consent prior to enrolment. People interested in participating will be met by researchers at the study site to obtain consent and complete the baseline questionnaires.

Randomisation, allocation concealment and sequence generation

Data collected from the screening visit will be utilised to assign participants to groups based on sex and BMI in the process of randomisation by minimisation. This process will ensure the baseline characteristics are balanced between the treatment groups and have been utilised as a reasonable method for randomisation for small clinical trials to reduce bias. Participants will be randomised at an individual level by an independent statistician without contact with participants during the trial. Using a computer-based random number-producing algorithm, allocation sequence within will be generated to allow equal ratio of 1:1 to either the intervention or control group. Complete separation will be achieved between the statistician who generated the randomisation sequence and those involved in assessing participants and performing data entry.

It will not be possible to 'mask' researchers or participants to group allocation. However, those responsible for analysis will be masked to group allocation. A research assistant who will not be involved in the enrolment, assessment or allocation of participants will pack and sequence

For peer review only - http://bmjopen.bmj.com/site/about/guidelines.xhtml

pre-packed envelopes with the group allocation. Only after the study investigator/ sub-investigator reviews the eligibility based on health records, laboratory results and concomitant medications, and confirms eligibility to proceed, will the envelope be opened and details of the particular study group will be revealed to the participant. If the participant is randomised into the intervention (INT) group, layperson client printouts and summary will then be provided with their program resources.

Sample Size Calculation

The sample size calculation was based upon retrospective dataset from the SWMS (25), with a derived standard deviation of 4kg. Thus, 42 participants in each group will give our study 80% power to detect a difference in weight change between groups of 3kg at the 5% significance level using a two-sided test. A between-group difference of 3kg was chosen as this is outside the range of normal weight fluctuation and is sufficient to sustain clinically meaningful weight loss (5). Accounting for attrition rate of 20%, the total target recruitment is 105 participants.

Study Intervention

The intervention (INT) group participants will receive EE information generated from ECAL IC which will encompass the REE and RQ delivered in the form of a layperson client report containing summary of recommendations to help improve understanding about metabolic health (example of client report can be found in supplementary material in Appendix). During the study visit, these results will serve as a reference tool for the dietitian when formulating a dietary plan based on the measured REE to determine total daily EE (TDEE). Further, the RQ data will also be used as an indicator of substrate oxidation to deliver key messages on carbohydrate vs. fat oxidation and to facilitate decision making in energy-restricted dietary

For peer review only - http://bmjopen.bmj.com/site/about/guidelines.xhtml

recommendations. The clinician will explain the measured REE (supposedly more accurate than predicted REE) in the context of weight loss and make recommendations on dietary plan e.g. energy restrictions based on measured REE. Food diaries will be provided to study participants to support and check compliance and used to record compliance with recommendation. The threshold of compliance with study intervention is set at those who complete at least 5 out of the 9 study visits from baseline to final visit with completion of the IC test. The energy requirements of each participant in the INT group will be calculated using the measured REE and the self-reported physical activity captured via the International Physical Activity Questionnaire (IPAQ). The recommended energy restriction for weight loss will be up to 30% less than total daily energy requirement to achieve the intended weight loss. The individualized weight loss plans will be modified at study visits based on the REE and RQ information and the weight loss plans adapted if more energy restriction is required. The dietitians will utilise standardized diet checklists to assist with monitoring dietary compliance and cross-check with the self-completed food diaries. We have set the threshold of compliance with dietary recommendations at 50% compliance.

In comparison, the participants in the standard care (SC) or control group will receive usual care delivered via multidisciplinary weight management intervention including dietary intervention, recommendations of physical activity and behaviour modification. Dietary restrictions will be estimated based upon the Harris-Benedict equation for total daily energy requirements, with a recommended 30% energy deficit from total daily energy requirement. Typically, suggested meal plans range from approximately 1,200 to 2,500 kcal per day. Dietetic advice will be based upon the Association of UK dieticians (BDA)(26) recommendation on daily intake of carbohydrates (40-60%), protein (20-30%), and fat (20-30%). Participants will be encouraged not to have prolonged episodes of fasting, but to

For peer review only - http://bmjopen.bmj.com/site/about/guidelines.xhtml

maintain up to three regular portioned balanced meals/day, while limiting snacks and reinforcing portion control. Dietitian will provide the participant with a food diary and offer them self-monitoring tools to monitor diet using MyFitnessPal™ Mobile App (www.myfitnesspal.com) for at least three days per week. Up to 150 minutes of physical activity/week will be recommended. Participants will be encouraged to formulate personalised dietary restriction and achievable activity goals each week during each study visit. In order to make a comparison of indirect measurements of REE and RQ between groups, the participants receiving SC will also undergo IC measurement, but without receiving the EE information. For full details on assessments during study visits, refer to Table 2.

Study Outcomes

Primary Outcome

The primary outcome is the difference in change of weight, in absolute value (kg), between participants in the INT group (EE information plus SC) versus the SC group at baseline and 24-weeks after randomisation.

Secondary Measurements and Outcomes

Anthropometrics, weight change, body composition (fat mass, fat-free mass), obesity-associated comorbidities, changes in REE, RQ and substrate (fat and glucose) oxidation based on IC, will be measured and determined throughout the study.

Data Collection

The following section outlines the data and biochemical evaluations being collected during the test periods (see Table 2 for a summary).

For peer review only - http://bmjopen.bmj.com/site/about/guidelines.xhtml

Anthropometry

Assessment of anthropometric data will be obtained at screening, baseline, week 4, 8, 12, 16, 20 and 24 visits. Body weight will be measured and recorded to the nearest 100g following an overnight fast and will be captured twice on each occasion. The weighing scale used will be the same scale which will be calibrated and used throughout the study. Height will be measured twice to the nearest 1 mm with the average value taken and recorded from a stadio-meter at the screening visit. BMI will be determined as weight/height squared (kg/m^2). Body composition will be determined using the two-electrode leg-to-leg bio-impedance analyser machine (Tanita TBF-300MA, Tanita Corporation, Tokyo, Japan) and the average value calculated. Participants will wear a light gown, and all external metal objects will be removed prior to measurement. Total body fat mass (%, kg), total body lean mass (%, kg) will be obtained. Waist circumference will be measured to the nearest 1 mm, using a measuring tape at the mid-point between the lower costal border and the iliac crest with the average of 3 measurements taken. In addition to the baseline anthropometric measurements and end of weight loss intervention (week 24), weight will also be recorded during dietetic visits and counselling to provide feedback to participants in both groups. Regular weight monitoring allows the study clinicians and participants to assist with weight monitoring, associated with improved success and compliance to weight loss intervention.

Biochemical measures

At screening, baseline, and end of study (week 24) visits, fasting (>8hours) venous blood samples will be collected by study clinician. Collected blood samples will be centrifuged (at 4°C, 4000 rpm, 10 min) to separate plasma or serum and stored at -80°C for subsequent analysis.

For peer review only - http://bmjopen.bmj.com/site/about/guidelines.xhtml

ECAL IC Protocol

Prior to each IC measurement, all participants will receive detailed preparatory oral and printed information. Participants will be asked to abstain from food, alcohol, and any calorie-containing beverages for at least 8 hours and refrain from vigorous physical activity for at least 12 hours prior to the measurement. Participants will arrive between 10 – 12 hours after an overnight fast before undergoing the IC test. Participants will be asked to confirm their fasting duration and will be reminded to follow preparatory procedures prior to next visit. The ECAL indirect calorimeter (Metabolic Health Solutions Pty Ltd, Australia) used will be calibrated using the 5% carbon dioxide to calibrate gas flow, as per manufacturer specifications. Anthropometric data will be entered into the EC Health software linked up to the ECAL IC. Participant will be lying comfortably in a supine position and elevated at a 45-degrees angle. After 15 minutes, a nose clip will be applied over the nostrils and participant will be instructed to breathe through a single-use mouthpiece, ensuring a tight seal over it. The test instructor will observe the volumetric flow and ensure that a steady volume and rate of breathing is maintained for between 8 to 10 minutes. A successful test will be defined as a steady-state achieved with a minimum of 5 consecutive minutes with less than 10% coefficient of variation in FEO_2 and $FECO_2$ (27). The instructor will stop the indirect calorimetry measurement when this steady-state has been achieved. Should any interruptions occur or failing to achieve a steady-state, a repeat testing will be performed.

Client Report

A client report will be generated using the EC Health software when the IC measurement is complete. A series of questions related to their time-specific metabolism will be completed and a client report generated for participant and clinician in the intervention group only. An example of client report can be found in Supplementary Material, Appendix.

For peer review only - http://bmjopen.bmj.com/site/about/guidelines.xhtml

Resting Energy Expenditure

All participants including the SC and INT group will undergo ECAL IC after an overnight fast at baseline, week 2, 4, 8, 12, 16, 20 and 24 visits, using a mouthpiece whilst in a supine and rested state. The ECAL IC will be calibrated as described within the ECAL IC protocol section. All ECAL IC testing will be conducted in the morning in a temperature-neutral environment with participants lying in a comfortable supine position. During the measurement duration of between 8-10 minutes, participants will be asked to remain as relaxed as possible without falling asleep and instructed not to speak or fidget. The VO_2 and VCO_2 will be continuously measured for 8-10 minutes. After discarding the first 2 min of data, REE will be calculated as the lowest consecutive 5 min average value, provided that the coefficient of variation within that 5 min interval is <10%. The ECAL IC will calculate the REE based on the Weir equation (28). This information will be utilized by the dietitian to formulate a dietary plan and make recommendations of an energy-restricted diet for participants randomised to the INT group. In contrast, participants and dietitians providing care within the SC group will not receive this information but dietary estimate of total daily EE will be based upon the Harris-Benedict predictive EE equation.

Respiratory Quotient

Similarly, the ECAL IC testing conducted at baseline, week 2, 4, 8, 12, 16, 20 and 24 visits, will generate an RQ value alongside the REE. The IC will analyse the respiratory gas exchange of the participants to determine VO2 and VCO2 and the ratio of VCO2 to VO2 (VCO2/VO2), will determine the RQ. RQ will be utilised as an indicator of measurement adequacy and of substrate oxidation. Based on the gas exchange dataset, the ECAL IC will generate information on carbohydrate oxidation (%) and fat oxidation (%). This information

For peer review only - http://bmjopen.bmj.com/site/about/guidelines.xhtml

will be utilized by the dietitian to formulate a dietary plan and make recommendations of an energy-restricted diet for participants randomised to the INT group.

Dietary Analysis

Participants will be provided instructions on completing the 3-day food diary. Participants will be provided with cups, spoons, or utilise a food scale to accurately determine food portion sizes. Participants will be asked to record estimate volumes and portion sizes. Issues regarding estimating portion sizes and measuring foods will be discussed. Participants will be informed to record two week days and one weekend day before their next study visit and were told to provide exact brand names where possible. Participants will be encouraged to select days when their normal routine was least likely to be disrupted and were instructed to record meals eaten away from the home to the best of their ability. All food diaries will be checked by the study dietitians for missing data or obvious errors in recording. Adherence to the energy-restricted dietary recommendations (30% below total daily EE) will be assessed using the food diary 3 x 24 hour dietary recalls together with the dietician at study visit (baseline, week 4, 8, 12, 16, 20, and 24). The threshold of compliance to recommended dietary restriction based on self-reported food diary will be set at >80%. All participants will be asked to return the food diary and provided a reminder to bring along the subsequent food diary at the following visit.

Activity Energy Expenditure

Physical activity will be evaluated based upon the self-reported International Physical Activity Questionnaire (IPAQ) long form (29) at baseline, week 12 and week 24. Participants will be asked to record the duration and frequency of mild-, moderate- and vigorous-intensity activity levels within the past 7 days. The hours spent in sleep and mild-, moderate- and vigorous-

For peer review only - http://bmjopen.bmj.com/site/about/guidelines.xhtml

activity were multiplied by respective metabolic equivalents(METs), summed and finally expressed as total MET-h/week. The data on physical activity from self-reported IPAQ captured will be used to calculate energy recommendation for weight loss.

Quality of Life Assessment

Assessment of quality of life outcome measures will be conducted via the IWQoL-Lite and EQ-5D-5L questionnaires. Participants will complete these questionnaires at baseline and final visit (week 24).

Meal Test

All participants will be offered the option of participating in a meal test consisting of 35% of the participant's calculated 24-hour energy requirement in the form of the Ensure Plus high-caloric milkshake. After an overnight fast of at least 8 hours, participants will be offered Ensure Plus milkshake which consists of a 220mls of milkshake style oral nutritional supplement with energy content of 1.5kcal/ml. Ensure Plus consist of 53.8% calories from carbohydrate, 29.5% from fat and 16.7% from protein. Participants will be given up to 5 minutes to consume these high-caloric milkshakes worth 35% of their total daily energy needs. The majority of participants will be having to consume less than 500 ml of high-caloric milkshake. Blood samples will be taken to determine baseline concentrations of plasma glucose, insulin and appetite-related gut hormones including glucagon-like peptide-1 (GLP-1), gastric inhibitory polypeptide (GIP) and peptide YY (PYY). Blood samples will then be taken at 15, 30, 60, 90 and 120 minutes after the meal ingestion.

Glycaemic Variability

For peer review only

For peer review only - http://bmjopen.bmj.com/site/about/guidelines.xhtml

We will investigate the secondary impact of weight loss on glycaemic variability in a subset of obese individuals with prediabetes and compare the changes in mean amplitude of glycaemic excursion (MACE) using Continuous Glucose Monitoring (CGM). We aim to recruit up to 15 participants with prediabetes, defined as glycated haemoglobin HbA1c of 42 – 47 mmol/mol and an equal representation of another 15 patients without diabetes to undergo this sub-group study.

Data management

All study participants will be given a unique identifier which will be utilized to identify their electronic and paper-trail data and biochemical lab samples. The database will be stored on a password-protected computer, accessible to the study researchers only, containing participant identifiers and their associated unique identifier. Paper based data will be stored securely at the Research & Development Centre, Clinical Sciences Building, University Hospital Aintree, Liverpool, United Kingdom for 15 years, after which it may be destroyed. Case report forms will be utilised to compile and collect data at all study visits. Before analysis, data will be compared between files to ensure accuracy of transcription. Biological samples including blood tests will be stored in a secure swipe card access secured -80°C freezer, with a temperature logbook and alarm system that alerts a staff member should temperature rise above a predetermined set range. Every sample stored in the freezer will be recorded in the logbook of biological samples. Samples will be stored for up to 15 years from collection date and disposed of accordingly after that time.

Data Monitoring

The study will not have a formal data monitoring committee as adverse events of treatments are well known within weight loss interventions within a specialist healthcare setting. Any

For peer review only - http://bmjopen.bmj.com/site/about/guidelines.xhtml

unexpected serious adverse events or outcomes (such as incapacitation or death) will be discussed by the trial management committee (identical to the authors of this protocol). Furthermore, the trial management committee will monitor recruitment, treatment and attrition rates and any concerns related to the study.

Protocol deviations

Deviations from proposed study protocol will be communicated to the study sponsor at University of Liverpool / Liverpool Joint Research Office and the Research & Development Unit based at University Hospital Aintree, Liverpool.

Adverse events

Adverse events will be captured onto the case report forms and will be reported to the University of Liverpool / Liverpool Joint Research Office and the Research & Development Unit based at University Hospital Aintree, Liverpool. Adverse events that lead to participant withdrawals will be reported in any future publications. We do not intend to formally analyse adverse events.

STATISTICAL ANALYSIS PLAN

Statistical analysis will be performed using IBM SPSS Statistics for Windows V.24.0 or later. All variables will be checked for plausibility and missing values. Outcome data will be presented as mean (SD) for continuous variables and counts (percentages) for categorical variables. The characteristics of completers will be tested using independent *t*-tests for between group analyses and paired t-test for within group analyses. A non-parametric counterpart will be used if data are not normally distributed. Chi-squared (χ^2) tests will be utilised for categorical variables. The primary analysis will utilise linear mixed models to

For peer review only - http://bmjopen.bmj.com/site/about/guidelines.xhtml

assess impact of intervention (EE information plus SC vs. SC alone) on the net change in body weight (baseline, week 24), in absolute terms (kg) between both groups. The covariates age, sex and BMI will also be included in the primary analysis model. Both intention-to-treat (ITT) and per-protocol analyses (for those who achieve a minimum of 5 out of the 9 study visits with completion of IC tests as defined in the 'study intervention') will be completed. Where main effects are identified, Bonferroni post hoc tests will be performed to identify significant differences between means (a significant p-value set at <0.05). While the ITT analysis will be the main analysis, the per-protocol analysis will allow us to determine that the true efficacy of the intervention for participants who strictly adhered to the protocol.

The secondary analyses will include change of body composition (body fat %, fat-free mass), change in REE, change in RQ with time (treated as categorical with levels at baseline, 4, 8, 12 and 24 weeks), and treatment-by-time interaction. We did not undertake formal sample size calculation for secondary analysis as the study was powered to the primary endpoint only. As a result, this will provide data as exploratory analysis only. Type I error will be modified by utilizing Bonferroni adjustment or the equivalent non-parametric test. Age, body mass index, fat-free mass, obesity-related comorbidities (King's Obesity Staging Criteria), quality of life outcomes and glycaemic variability will be examined to determine whether they contribute significantly to the models. If a covariate is significant, a term will be added to the model to adjust for the effects and two-way interactions with time and treatment will also be examined. If these interactions are also significant they will be similarly adjusted for in the model using analysis of covariance (ANCOVA).

Data access

There are no contractual agreements that require the data from this trial to be shared.

For peer review only - http://bmjopen.bmj.com/site/about/guidelines.xhtml

ETHICS AND DISSEMINATION

Ethics approval was obtained from the Health Research Authority and the North West Research Ethics Committee (18/NW/0645) and is conducted in accordance with the Declaration of Helsinki and Good Clinical Practice (GCP). Participants will also receive a copy of their individual results including anthropometric data, body weight change and results of EE and RQ from ECAL IC tests. They will also be provided with a lay summary of overall results of the study. Findings from this study will be disseminated at relevant scientific conferences and the data published in research manuscripts in peer-reviewed journals.

DISCUSSION

The study goal is to assess the impact of utilising REE and RQ from IC as an adjunctive management for weight loss. The role of utilising EE information to influence behaviour has yet to be evaluated within a real-world pragmatic RCT. Further, the results will help advance existing evidence about the relationship between RQ, REE and change in body weight.

Several experimental studies evaluating change in REE as a response to overfeeding (i.e. 40-70% increase from baseline energy requirements) suggested that short-term (2-8 weeks) overfeeding produces less weight gain than expected from the controlled increase in intake, due to greater increase in REE (30-34). Such compensatory increase in energy intake lead to weight increases, of which 55-67% constitutes fat mass gain (30, 31, 35, 36). Conversely, underfeeding studies reveal that in short-term, intentional weight loss leads to decrease in EE beyond predicted values(37-39). Decrease in REE is disproportionate to weight measured per kilogram against FFM after weight loss. The regression analysis of change in REE against the change in FFM reveals that decline in REE tended to be greater than

For peer review only - http://bmjopen.bmj.com/site/about/guidelines.xhtml

accounted for by loss of FFM. Such over-compensatory metabolic changes act to oppose further weight change (40).

Variability in substrate oxidation between individuals may be an inherent variability that contributes towards weight regain and fat storage. Variability of macronutrient composition intake between participants may influence the measured RQ and influence the results associated with fat vs. carbohydrate oxidation(41). Several studies have suggested macronutrient alterations could also influence appetite-regulation (42, 43). Further, individual differences in circulating insulin levels or sensitivity may confound the reported associations between RQ, REE and fat mass(14, 44, 45). We have taken this into account and have therefore excluded patients with diabetes mellitus or metabolic disorders.

IC use has been validated through several detailed experimental in vitro and in vivo studies, demonstrating accuracy of measuring EE when compared to the mass spectrometry and Douglas Bag method (46). Given that previous studies on other commercial metabolic monitors showed variations in gas exchange rates (47), leading to secondary metabolic results variability, participants will be invited to strictly adhere to the protocol requirements and all measurements performed under controlled and steady-state experimental setting.

Strengths

There are several strengths to the study methodology, in particular the randomized controlled trial design. This will be the first RCT to assess whether providing EE and RQ from ECAL IC will influence the outcome of weight loss intervention when integrated with communication between the clinician and the patient. The study is powered statistically to evaluate weight change within the primary analysis model. The study has the potential to expand our

For peer review only - http://bmjopen.bmj.com/site/about/guidelines.xhtml

understanding of how EE information can be integrated into communication and behaviour modification aspects of weight management clinics, while providing assessment of detailed secondary outcome measures including but not limited to body composition, EE, substrate oxidation, quality of life outcome measures. Further, the secondary analysis provides a comparison between the measured REE from IC with that of predicted REE from Harris-Benedict equation which serves to enhance our understanding of accuracy and practicality of use of ECAL IC to help determine weight change in obesity. One of the challenges with any weight management study is to maintain high levels of motivation and engagement. We have been successful in recruiting large number of participants for similar weight loss intervention trials and plan to provide support for participants with routine appointments with the dietician and multidisciplinary weight management team.

Limitations

Whilst the ECAL IC is purpose-built as a portable, convenient and practical device designed to capture EE information in users over a relatively brief time period, ECAL IC provides a wider degree of variation and greater limits of acceptability in the repeatability of the REE, RQ and substrate oxidation measures during steady state when the device is compared against other IC machines using Deltatrac as the standard reference method (8). In addition, the measured REE will be influenced by age, sex, body size and variation in individual physical activity levels (48, 49). Under strict controlled research lab environments with overnight fasting, the EE information obtained are accurate within the time constraints of those settings (steady state & fasting) only, but may not reflect the day-to-day fluctuations in the assessment of energy cost of participants due to energy cost of physical activity, change in energy intake and macronutrient dietary composition (50). The utility of IPAQ as a self-reported indicator to determine physical activity level in people with obesity showed a weaker

For peer review only - http://bmjopen.bmj.com/site/about/guidelines.xhtml

correlation, and tended to overestimate activity levels when compared against reference standards using an accelerometer or pedometer (51). A recent systematic review reported that increased exercise and activity levels, including short-term moderate- to high-intensity exercise training was associated with only modest changes in body composition (52). The reliance on bioelectrical impedance analysis to capture data on body composition may result in a wide degree of variation in capturing data on fat-mass and FFM when compared against the use of body densitometry. This study is designed to evaluate the impact of intervention during the 24-week duration but does not specifically evaluate the impact of providing EE information during the weight maintenance phase after the initial weight loss.

This 6-month RCT will provide practical feedback on acceptability and applicability of EE and RQ measured from the portable ECAL IC to facilitate weight loss strategies. This may lead to incorporating strategies for weight loss using EE and RQ as basis of dietetic advice and recommendations which will inform the design of future weight management trials.

For peer review only - http://bmjopen.bmj.com/site/about/guidelines.xhtml

Author affiliations

1 Department of Cardiovascular and Metabolic Medicine, Institute of Life Course and Medical Sciences, University of Liverpool, UK.

2 Department of Diabetes & Endocrinology, University Hospital Aintree, Liverpool University Hospitals NHS Foundation Trust, Liverpool, UK.

Contributions

The study chief investigators J.Z.M.L., U.A., D.J.C., and J.P.H.W were responsible for creating the research question, design of the study, obtaining ethical approval, the acquisition of funding and oversight to study implementation. All authors were responsible for the drafting of this manuscript.

Acknowledgements

Professor J.P.H.W. and U.A. were primary supervisors for J.Z.M.L. in conduct of this study contributing towards completion of PhD.

Funding

This work was funded by the University of Liverpool (UoL001379). The ECAL indirect calorimeter was provided and maintained by the manufacturer Metabolic Health Solutions, Australia.

Disclaimer

The funding source and funding bodies did not have any input into the design of the study, the collection or analysis of data, the preparation of this manuscript or the decision to submit this manuscript for publication.

For peer review only

Competing interests

J.P.H.W. reports personal fees, grants, and consultancy fees paid to his institution from AstraZeneca, Novo Nordisk, and Takeda, personal fees and consultancy fees paid to his institution from Boehringer Ingelheim, Janssen, Napp, Mundipharma, and Sanofi, and consultancy fees paid to his institution from Lilly, Rhythm Pharmaceuticals and Wilmington Healthcare outside of the submitted work.

J.Z.M.L., U.A., D.J.C. has no competing interests to declare.

Patient and Public Involvement

Patients were involved in the design and conduct of this research during the feasibility stage, priority of the research question, choice of outcome measures, and methods of recruitment. Refer to the Methods section for further details.

For peer review only

Table 1: Inclusion and exclusion criteria from protocol

Inclusion criteria

- Man or woman, 18 to 70 years of age
- BMI ≥30 kg/ m^2 to ≤ 60 kg/m^2 at screening visit
- Stable weight (change of < 5% within 12 weeks before screening based on medical history)
- Subjects are in the investigators opinion, well-motivated, capable, and willing to learn how to undergo indirect calorimetry testing, as required for study
- Willing and able to adhere to the prohibitions and restrictions specified within this protocol

Exclusion criteria

- Taking weight loss medication within 12 weeks prior to randomisation
- Previous or planned bariatric surgery
- History of type 1, type 2 diabetes mellitus, diabetic ketoacidosis, or diabetes secondary to pancreatitis
- Has a HbA1c of ≥ 6.5% (or ≥ 48 mmol/mol)
- History of obesity with a known secondary cause (e.g. Cushing's disease / syndrome)
- Oral corticosteroid use (except in the short term use of a 7-10 day course)
- Ongoing, inadequately controlled thyroid disorder defined as thyroid-stimulating hormone >6mIU/litre or <0.4 mIU/litre
- History of malignancy within 3 years before screening (or diagnosis of malignancy within this period)
- eGFR ≤30ml/min/1.73m^2 on serum testing
- Alanine aminotransferase level is >2.0 times the upper limit of normal or total bilirubin is >1.5 times the upper limit of normal at screening
- Other major illness likely to preclude participation in the trial
- History of glucagonoma
- A myocardial infarction, unstable angina, revascularization procedure (stent or bypass graft surgery) or cerebrovascular accident within 12 weeks before screening
- Heart Failure NYHA Class III-IV

For peer review only - http://bmjopen.bmj.com/site/about/guidelines.xhtml

- End-stage Chronic Obstructive Pulmonary Disease (COPD)

Withdrawal Criteria

- Terminal illness or loss of capacity during participation in clinical trial
- In the opinion of the investigator is unsafe for continuation in study for medical, safety, regulatory or other reasons
- Loss to follow-up
- Female participants with a positive pregnancy test

BMI, Body mass index; HbA1c, glycosylated haemoglobin; eGFR, estimated glomerular filtration rate; NYHA, New York Heart Association.

For peer review only - http://bmjopen.bmj.com/site/about/guidelines.xhtml

Table 2: Assessments and procedures during study visits.

**Procedure** Visit window(days)	SCR ±7	W0	W1 ±3	W2 ±3	W4 ±3	W8 ±3	W12 ±3	W16 ±3	W20 ±3	W24 ±3
**Visit**	**1**	**2**	**3**	**4**	**5**	**6**	**7**	**8**	**9**	**10**
Informed consent	X									
Inclusion exclusion criteria	X	X								
Randomisation criteria and randomisation		X								
Medical History	X									
Physical examination	X									
Indirect calorimetry		X	X	X	X	X	X	X	X	X
Full blood count	X									
Renal profile	X									
Lipid profile		X								X
Liver function test	X									X
Thyroid function	X									
GLP1, GIP, PYY		X								X
Fasting glucose		X								X
HbA1c	X									X
Urine pregnancy test	X									
Height	X									
Weight	X	X	X	X	X	X	X	X	X	X
Waist, hip, thigh circumference		X		X	X	X	X	X	X	X
FFM, FM		X		X	X	X	X	X	X	X
Blood pressure	X	X			X	X	X	X	X	X
Diet counselling		X			X	X	X	X	X	X
Food Diary		X	X	X	X	X	X	X	X	X
Compliance check			X	X	X	X	X	X	X	X
IPAQ		X				X		X		X
Meal Test		X								X
CGM		X			X		X			X
Concomitant medication check	X	X	X	X	X	X	X	X	X	X
AE reporting		X			X	X	X	X	X	X
KOSC	X									X
ESS	X									X
STOP-BANG	X									X
IWQoL-Lite		X								X
EuroQoL-5D-5L		X								X

For peer review only

For peer review only - http://bmjopen.bmj.com/site/about/guidelines.xhtml

Acceptability survey	X	X

SCR – Screening visit; W0 – Week 0; W1 – Week 1; W2 – Week 2; W4 – Week 4; W8 – Week 8; W12 – Week 12; W16 – Week 16; W20 – Week 20; W24 – Week 24

AE – Adverse Events Reporting; CGM – Continuous Glucose Monitor; ESS, Epworth sleep score; EuroQoL-5D-5L – Questionnaire for Quality of life; FFM – Fat-free mass; FM – Fat mass; IPAQ International Physical Activity Questionnaire; IWQoL-Lite- Questionnaire for quality of life; KOSC, King's obesity staging score; STOP-BANG – questionnaire for sleep apnoea assessment;

For peer review only

References

1. Ng M, Fleming T, Robinson M, Thomson B, Graetz N, Margono C, et al. Global, regional, and national prevalence of overweight and obesity in children and adults during 1980–2013: a systematic analysis for the Global Burden of Disease Study 2013. The Lancet. 2014;384(9945):766-81.

2. World Health Organisation. Global Action for the Prevention and Control of NCDs 2013-2020 2013 [Available from: https://www.who.int/nmh/events/ncd_action_plan/en/.

3. Hruby A, Hu FB. The Epidemiology of Obesity: A Big Picture. Pharmacoeconomics. 2015;33(7):673-89.

4. Wing RR, Phelan S. Long-term weight loss maintenance. Am J Clin Nutr. 82. United States2005. p. 222S-5S.

5. Wing RR, Lang W, Wadden TA, Safford M, Knowler WC, Bertoni AG, et al. Benefits of Modest Weight Loss in Improving Cardiovascular Risk Factors in Overweight and Obese Individuals With Type 2 Diabetes. Diabetes Care. 2011;34(7):1481-6.

6. Sumithran P, Prendergast LA, Delbridge E, Purcell K, Shulkes A, Kriketos A, et al. Long-term persistence of hormonal adaptations to weight loss. N Engl J Med. 2011;365(17):1597-604.

7. Leibel RL, Rosenbaum M, Hirsch J. Changes in energy expenditure resulting from altered body weight. N Engl J Med. 1995;332(10):621-8.

8. Kennedy S, Ryan L, Fraser A, Clegg ME. Comparison of the GEM and the ECAL indirect calorimeters against the Deltatrac for measures of RMR and diet-induced thermogenesis. J Nutr Sci. 2014;3:e52.

9. Weir JB. New methods for calculating metabolic rate with special reference to protein metabolism. J Physiol. 1949;109(1-2):1-9.

10. Tissot S, Delafosse B, Bertrand O, Bouffard Y, Viale JP, Annat G. Clinical validation of the Deltatrac monitoring system in mechanically ventilated patients. Intensive Care Med. 1995;21(2):149-53.

11. Valtueña S, Salas-Salvadó J, Lorda PG. The respiratory quotient as a prognostic factor in weight-loss rebound. International Journal of Obesity. 1997;21(9):811-7.

12. Zurlo F, Lillioja S, Esposito-Del Puente A, Nyomba BL, Raz I, Saad MF, et al. Low ratio of fat to carbohydrate oxidation as predictor of weight gain: study of 24-h RQ. Am J Physiol. 1990;259(5 Pt 1):E650-7.

13. Toubro S, Sørensen TIA, Hindsberger C, Christensen NJ, Astrup A. Twenty-Four-Hour Respiratory Quotient: The Role of Diet and Familial Resemblance1. The Journal of Clinical Endocrinology & Metabolism. 1998;83(8):2758-64.

14. Galgani J, Ravussin E. Energy metabolism, fuel selection and body weight regulation. Int J Obes (Lond). 2008;32 Suppl 7(Suppl 7):S109-19.

15. Seidell JC, Muller DC, Sorkin JD, Andres R. Fasting respiratory exchange ratio and resting metabolic rate as predictors of weight gain: the Baltimore Longitudinal Study on Aging. Int J Obes Relat Metab Disord. 1992;16(9):667-74.

16. Filozof CM, Murúa C, Sanchez MP, Brailovsky C, Perman M, Gonzalez CD, et al. Low plasma leptin concentration and low rates of fat oxidation in weight-stable post-obese subjects. Obes Res. 2000;8(3):205-10.

17. Chaput JP, Pérusse L, Després JP, Tremblay A, Bouchard C. Findings from the Quebec Family Study on the Etiology of Obesity: Genetics and Environmental Highlights. Curr Obes Rep. 2014;3(1):54-66.

18. Goris AH, Westerterp KR. Postabsorptive respiratory quotient and food quotient-an analysis in lean and obese men and women. Eur J Clin Nutr. 2000;54(7):546-50.

For peer review only - http://bmjopen.bmj.com/site/about/guidelines.xhtml

19. Ellis AC, Hyatt TC, Hunter GR, Gower BA. Respiratory quotient predicts fat mass gain in premenopausal women. Obesity (Silver Spring). 2010;18(12):2255-9.
20. Marra M, Scalfi L, Contaldo F, Pasanisi F. Fasting Respiratory Quotient as a Predictor of Long-Term Weight Changes in Non-Obese Women. Annals of Nutrition and Metabolism. 2004;48(3):189-92.
21. Hainer V, Kunesová M, Parízková J, Stich V, Mikulová R, Slabá S. Respiratory quotient in obesity: its association with an ability to retain weight loss and with parental obesity. Sb Lek. 2000;101(1):99-104.
22. Jéquier E, Schutz Y. Long-term measurements of energy expenditure in humans using a respiration chamber. Am J Clin Nutr. 1983;38(6):989-98.
23. Saltzman E, Roberts SB. Effects of energy imbalance on energy expenditure and respiratory quotient in young and older men: a summary of data from two metabolic studies. Aging (Milano). 1996;8(6):370-8.
24. Hall KD, Bain HL, Chow CC. How adaptations of substrate utilization regulate body composition. Int J Obes (Lond). 2007;31(9):1378-83.
25. Steele T, Narayanan RP, James M, James J, Mazey N, Wilding JPH. Evaluation of Aintree LOSS, a community-based, multidisciplinary weight management service: outcomes and predictors of engagement. Clin Obes. 2017;7(6):368-76.
26. BDA Obesity Specialist Group. Dietetic Obesity Management Interventions in Adults: Evidence Review & Clinical Application.2018:[1-102 pp.].
27. Popp CJ, Butler M, Curran M, Illiano P, Sevick MA, St-Jules DE. Evaluating steady-state resting energy expenditure using indirect calorimetry in adults with overweight and obesity. Clinical Nutrition. 2019.
28. Weir JBdV. New methods for calculating metabolic rate with special reference to protein metabolism. The Journal of Physiology. 1949;109(1-2):1-9.
29. Levy SS, Readdy RT. Reliability of the International Physical Activity Questionnaire in Research Settings: Last 7-Day Self-Administered Long Form. Measurement in Physical Education and Exercise Science. 2009;13(4):191-205.
30. Ravussin E, Schutz Y, Acheson KJ, Dusmet M, Bourquin L, Jéquier E. Short-term, mixed-diet overfeeding in man: no evidence for "luxuskonsumption". Am J Physiol. 1985;249(5 Pt 1):E470-7.
31. Norgan NG, Durnin JV. The effect of 6 weeks of overfeeding on the body weight, body composition, and energy metabolism of young men. Am J Clin Nutr. 1980;33(5):978-88.
32. Dériaz O, Fournier G, Tremblay A, Després JP, Bouchard C. Lean-body-mass composition and resting energy expenditure before and after long-term overfeeding. Am J Clin Nutr. 1992;56(5):840-7.
33. Bray GA, Smith SR, de Jonge L, Xie H, Rood J, Martin CK, et al. Effect of dietary protein content on weight gain, energy expenditure, and body composition during overeating: a randomized controlled trial. Jama. 2012;307(1):47-55.
34. Leaf A, Antonio J. The Effects of Overfeeding on Body Composition: The Role of Macronutrient Composition - A Narrative Review. Int J Exerc Sci. 2017;10(8):1275-96.
35. Johannsen DL, Tchoukalova Y, Tam CS, Covington JD, Xie W, Schwarz JM, et al. Effect of 8 weeks of overfeeding on ectopic fat deposition and insulin sensitivity: testing the "adipose tissue expandability" hypothesis. Diabetes Care. 2014;37(10):2789-97.
36. Roberts SB, Young VR, Fuss P, Fiatarone MA, Richard B, Rasmussen H, et al. Energy expenditure and subsequent nutrient intakes in overfed young men. Am J Physiol. 1990;259(3 Pt 2):R461-9.

For peer review only - http://bmjopen.bmj.com/site/about/guidelines.xhtml

37. Heshka S, Yang MU, Wang J, Burt P, Pi-Sunyer FX. Weight loss and change in resting metabolic rate. Am J Clin Nutr. 1990;52(6):981-6.
38. Van Gaal LF, Vansant GA, De Leeuw IH. Factors determining energy expenditure during very-low-calorie diets. Am J Clin Nutr. 1992;56(1 Suppl):224s-9s.
39. Buscemi S, Verga S, Caimi G, Cerasola G. Low relative resting metabolic rate and body weight gain in adult Caucasian Italians. Int J Obes (Lond). 2005;29(3):287-91.
40. Weyer C, Pratley RE, Salbe AD, Bogardus C, Ravussin E, Tataranni PA. Energy expenditure, fat oxidation, and body weight regulation: a study of metabolic adaptation to long-term weight change. J Clin Endocrinol Metab. 2000;85(3):1087-94.
41. Schutz Y. Abnormalities of fuel utilization as predisposing to the development of obesity in humans. Obes Res. 1995;3 Suppl 2:173s-8s.
42. Kozimor A, Chang H, Cooper JA. Effects of dietary fatty acid composition from a high fat meal on satiety. Appetite. 2013;69:39-45.
43. Blundell JE, Stubbs RJ, Golding C, Croden F, Alam R, Whybrow S, et al. Resistance and susceptibility to weight gain: individual variability in response to a high-fat diet. Physiol Behav. 2005;86(5):614-22.
44. Thalacker-Mercer AE, Ingram KH, Guo F, Ilkayeva O, Newgard CB, Garvey WT. BMI, RQ, diabetes, and sex affect the relationships between amino acids and clamp measures of insulin action in humans. Diabetes. 2014;63(2):791-800.
45. Camastra S, Bonora E, Del Prato S, Rett K, Weck M, Ferrannini E. Effect of obesity and insulin resistance on resting and glucose-induced thermogenesis in man. EGIR (European Group for the Study of Insulin Resistance). Int J Obes Relat Metab Disord. 1999;23(12):1307-13.
46. Graf S, Karsegard VL, Viatte V, Heidegger CP, Fleury Y, Pichard C, et al. Evaluation of three indirect calorimetry devices in mechanically ventilated patients: which device compares best with the Deltatrac II(®)? A prospective observational study. Clin Nutr. 2015;34(1):60-5.
47. Cooper JA, Watras AC, O'Brien MJ, Luke A, Dobratz JR, Earthman CP, et al. Assessing validity and reliability of resting metabolic rate in six gas analysis systems. J Am Diet Assoc. 2009;109(1):128-32.
48. Hopkins M, Gibbons C, Caudwell P, Hellström PM, Näslund E, King NA, et al. The adaptive metabolic response to exercise-induced weight loss influences both energy expenditure and energy intake. Eur J Clin Nutr. 2014;68(5):581-6.
49. Gibbons MRD, Henry CJK, Ulijaszek SJ, Lightowler HJ. Intra-individual variation in RMR in older people. British Journal of Nutrition. 2004;91(3):485-9.
50. Goran MI, Beer WH, Wolfe RR, Poehlman ET, Young VR. Variation in total energy expenditure in young healthy free-living men. Metabolism. 1993;42(4):487-96.
51. Lee PH, Macfarlane DJ, Lam TH, Stewart SM. Validity of the international physical activity questionnaire short form (IPAQ-SF): A systematic review. International Journal of Behavioral Nutrition and Physical Activity. 2011;8(1):115.
52. Wewege M, van den Berg R, Ward RE, Keech A. The effects of high-intensity interval training vs. moderate-intensity continuous training on body composition in overweight and obese adults: a systematic review and meta-analysis. Obes Rev. 2017;18(6):635-46.

For peer review only - http://bmjopen.bmj.com/site/about/guidelines.xhtml

RESTING METABOLIC PROFILE

Client Name: Participant No.
Date of Test: --/--/--

	Test Score	Normal Range	Optimal
Resting Metabolism (kcal)	2103	1564 - 1912	N/A
Resting Metabolism (kJ)	8805	6548 - 8005	N/A
Fat Burning (%)	90.4	48 - 83	80
Glucose Availability (%)	9.6	17 - 52	20
Efficiency (FEO2) (%)	15.62	16 - 17	< 15

Background

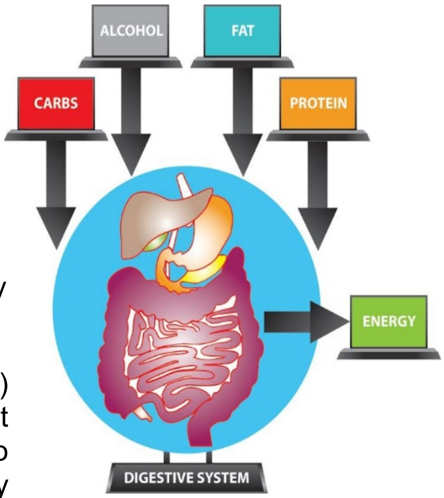

Energy production (metabolism) is vital to sustain every aspect of life and consequently is a key component of your health. For example, compromised energy production often results in an energy-related condition such as Obesity, Diabetes, Metabolic Syndrome, Chronic Fatigue, Insulin Resistance or a Sleep Disorder and may also be related to problems such hyperactivity, insomnia and an increased rate of aging.

Therefore understanding and optimising your metabolism is key to improving your health, weight management and wellbeing.

As humans we obtain energy from the food and liquids (fuel) consumed. The digestion of these nutrients and the subsequent absorption makes it possible for the mitochondria in cells to transform the potential chemical energy of food into the energy we use for life.

This energy is normally produced from a mixture of glucose (from carbohydrates) and fat. At rest and light exercise the body should produce around 80% of the required energy from fat stores. Under high intensity exercise the body gradually switches from burning fats to glucose. The ratio of these two fuels is highly individual and is determined primarily by the amount of glucose available, but also hormone function, fitness, muscle mass and general health.

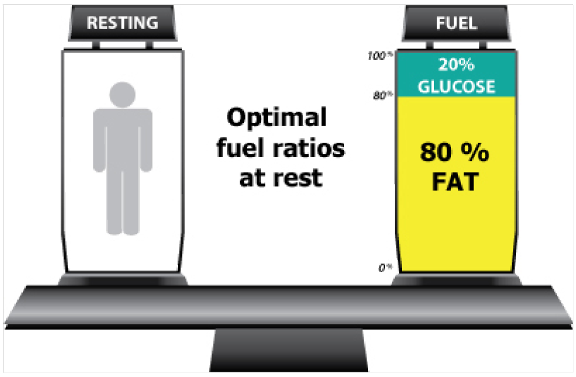

We have used a gold standard research technology called Indirect Calorimetry to measure your energy production. The device called ECAL allows our practitioners to accurately measure your daily energy requirement at rest, which nutrient source the energy is being produced from (fat and/or carbohydrate) and the efficiency with which your cells use oxygen.

Conventionally these energy requirements would be assessed using population based equations. However these are widely accepted as inaccurate because they cannot take into account individual variations in body composition, hormone function and physical activity levels. This can often lead to frustration for both client and practitioner.

For peer review only

Resting Energy Report of Participant

All of your test results are displayed in a table above, and where relevant normal or optimal values are also shown. However your metabolic profile is completely unique and therefore comparisons with these values are for general information only. Energy testing should be repeated over time and particularly following dietary or exercise intervention. This way you can measure the effectiveness of any strategy designed to improve your weight, energy and general health.

An explanation of each test score is provided and may include specific recommendations to optimize your energy production if necessary. Please consult with your health practitioner for appropriate diet, exercise or clinical intervention advice

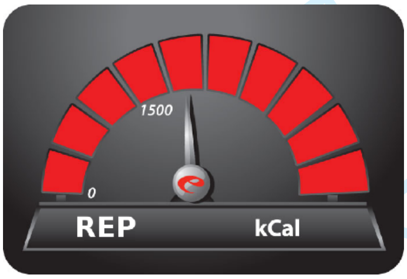

Resting Metabolism (REP/RMR) - This is the number of calories that would be expended over a 24 hour period if a person remained at rest/light activity. It usually represents around 65 - 80% of a person's total energy needs and is influenced by various factors including body composition, surface area, efficiency, age and hormones. ECAL accurately measures RMR and compares this against estimated values based on age, height, weight and sex.

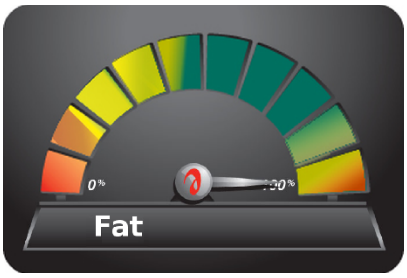

Fat Burning (%) - This score indicates the percentage of energy being generated from fat stores. The human body is designed to generate around 80% of total energy from fat stores at rest. This is particularly important for weight loss - to lose weight fat must be burnt. Factors that affect fat utilisation include diet, hormone function and physiological stress.

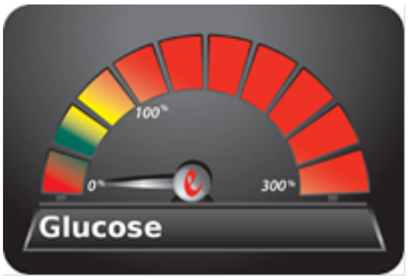

Glucose Availability (%) - This score indicates the amount of glucose (carbohydrate) currently available to generate energy. A surplus of glucose can result in elevated blood sugar levels, the storage of glucose and the conversion of glucose excess to fat, through a process known as lipogenesis.

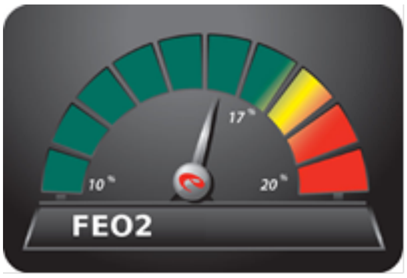

Efficiency (FEO2) - Our mitochondria (the part of the cell responsible for energy production) requires oxygen to convert the food we consume into useable energy. Healthier cells are more efficient and can therefore utilise more oxygen from the atmosphere - a lower value indicates more efficient mitochondria.

Recommendations to the Participant

RESTING Energy Expenditure

ECAL can accurately measure your REE and compare this against standard predictive equations.

High REE

A higher than expected resting energy level was recorded during the test.
Whilst high resting energy production generally results in better weight control it may be symptomatic of poor health. For example, high energy levels may cause symptoms such as hyperactivity, anxiety, insomnia, fatigue, low body weight and a difficulty maintaining muscle mass. The physiological reasons for recording a higher than normal resting energy level may include; (i) High mitochondrial function (FEO2 < 15%) (ii) Poor mitochondrial function (FEO2 > 17%) (iii) Fast breathing rate (RR > 15) (iv) Deep breathing (Ex Vol > 1.0L/breath)

RESPIRATORY QUOTIENT (RQ)

Normal RQ

A normal mean RQ score was recorded during the resting energy test.
It is common to read a normal RQ score and assume a state of good health, however the RQ score is calculated from two respiratory parameters; volume of expired carbon dioxide and the volume of oxygen consumed. It is therefore important to check that these two variables are also within normal range.

Are you having difficulty losing weight?

While RQ was measured as normal, weight control is dependent on other factors such as total calorie intake, insulin and the type of nutrients consumed.
It is very difficult to lose weight if one or all of the following conditions exist;
1. If the calorie intake is higher than the calorie expenditure.
2. If insulin is being released at rest.

Recommendations

Recommendations for 1:
a. Request the client accurately records their food and fluid intake over 5 - 7 consecutive days.
b. Calculate the client's average calorie intake per day and compare to their resting energy level. c. If the client's resting energy level is lower than their calorie intake, advise the client to increase their daily calorie expenditure by participating in moderate exercise for a minimum of 30 minutes per day and reduce calorie intake through reduced portion size.
Recommendations for 2:
a. Consider pathology testing of fasting insulin levels to confirm hyperinsulinaemia. b. Advise a reduction in the daily glucose intake.
c. Use the Glucose Calculator in the "Tools" section of ECHealth to calculate the client's glucose capacity and then prescribe 50 - 75% of the calculated liver capacity (grams) of glucose per day. Apply glucose restriction for 5 - 7 days.
d. Advise the client to participate in 20 - 30 minutes of moderate to intense exercise daily (as appropriate) to assist in lowering the high glucose level.
e. Repeat the resting energy test within 5 - 7 days to determine the effect of glucose restriction. f. If improved at the second test develop an eating plan based on 20% of the client's total daily energy expenditure (TEE = resting energy + energy expended through activity) to calculate the
amount of glucose required per day.
g. If no change advice the client to undertake pathology testing of the sex hormones and cortisol in the morning around 8am to exclude a hormonal imbalance.

For peer review only

Reporting checklist for protocol of a clinical trial.

Based on the SPIRIT guidelines.

Instructions to authors

Complete this checklist by entering the page numbers from your manuscript where readers will find each of the items listed below.

Your article may not currently address all the items on the checklist. Please modify your text to include the missing information. If you are certain that an item does not apply, please write "n/a" and provide a short explanation.

Upload your completed checklist as an extra file when you submit to a journal.

In your methods section, say that you used the SPIRITreporting guidelines, and cite them as:

Chan A-W, Tetzlaff JM, Altman DG, Laupacis A, Gøtzsche PC, Krleža-Jerić K, Hróbjartsson A, Mann H, Dickersin K, Berlin J, Doré C, Parulekar W, Summerskill W, Groves T, Schulz K, Sox H, Rockhold FW, Rennie D, Moher D. SPIRIT 2013 Statement: Defining standard protocol items for clinical trials. Ann Intern Med. 2013;158(3):200-207

		Reporting Item	Page Number
**Administrative information**			
Title	#1	Descriptive title identifying the study design, population, interventions, and, if applicable, trial acronym	1
Trial registration	#2a	Trial identifier and registry name. If not yet registered, name of intended registry	3, 8-9
Trial registration: data set	#2b	All items from the World Health Organization Trial Registration Data Set	8-9,19-22
Protocol version	#3	Date and version identifier	8
Funding	#4	Sources and types of financial, material, and other support	8-9,26-27
Roles and responsibilities: contributorship	#5a	Names, affiliations, and roles of protocol contributors	26-27

For peer review only

Roles and responsibilities: sponsor contact information	#5b	Name and contact information for the trial sponsor	8-9,22, 26-27
Roles and responsibilities: sponsor and funder	#5c	Role of study sponsor and funders, if any, in study design; collection, management, analysis, and interpretation of data; writing of the report; and the decision to submit the report for publication, including whether they will have ultimate authority over any of these activities	8-9, 19-20, 22, 26-27
Roles and responsibilities: committees	#5d	Composition, roles, and responsibilities of the coordinating centre, steering committee, endpoint adjudication committee, data management team, and other individuals or groups overseeing the trial, if applicable (see Item 21a for data monitoring committee)	8-9, 19-20, 22, 26-27
**Introduction**			
Background and rationale	#6a	Description of research question and justification for undertaking the trial, including summary of relevant studies (published and unpublished) examining benefits and harms for each intervention	4-8
Background and rationale: choice of comparators	#6b	Explanation for choice of comparators	7-8, 10-13
Objectives	#7	Specific objectives or hypotheses	7-8
Trial design	#8	Description of trial design including type of trial (eg, parallel group, crossover, factorial, single group), allocation ratio, and framework (eg, superiority, equivalence, non-inferiority, exploratory)	8
**Methods: Participants, interventions, and outcomes**			
Study setting	#9	Description of study settings (eg, community clinic, academic hospital) and list of countries where data will be collected. Reference to where list of study sites can be obtained	8-11

For peer review only

Eligibility criteria	#10	Inclusion and exclusion criteria for participants. If applicable, eligibility criteria for study centres and individuals who will perform the interventions (eg, surgeons, psychotherapists)	9-10
Interventions: description	#11a	Interventions for each group with sufficient detail to allow replication, including how and when they will be administered	11-13
Interventions: modifications	#11b	Criteria for discontinuing or modifying allocated interventions for a given trial participant (eg, drug dose change in response to harms, participant request, or improving / worsening disease)	11-13,20
Interventions: adherance	#11c	Strategies to improve adherence to intervention protocols, and any procedures for monitoring adherence (eg, drug tablet return; laboratory tests)	11-13, 19-20
Interventions: concomitant care	#11d	Relevant concomitant care and interventions that are permitted or prohibited during the trial	11-13, 19-20
Outcomes	#12	Primary, secondary, and other outcomes, including the specific measurement variable (eg, systolic blood pressure), analysis metric (eg, change from baseline, final value, time to event), method of aggregation (eg, median, proportion), and time point for each outcome. Explanation of the clinical relevance of chosen efficacy and harm outcomes is strongly recommended	13-20, 30-31
Participant timeline	#13	Time schedule of enrolment, interventions (including any run-ins and washouts), assessments, and visits for participants. A schematic diagram is highly recommended (see Figure)	9-10, 30-31
Sample size	#14	Estimated number of participants needed to achieve study objectives and how it was determined, including clinical and statistical assumptions supporting any sample size calculations	10-11
Recruitment	#15	Strategies for achieving adequate participant enrolment to reach target sample size	10-11, 19-20
**Methods: Assignment of interventions (for controlled trials)**			
Allocation: sequence generation	#16a	Method of generating the allocation sequence (eg, computer-generated random numbers), and list of any factors for stratification. To reduce predictability of a random sequence,	10-11

For peer review only

		details of any planned restriction (eg, blocking) should be provided in a separate document that is unavailable to those who enrol participants or assign interventions	
Allocation concealment mechanism	#16b	Mechanism of implementing the allocation sequence (eg, central telephone; sequentially numbered, opaque, sealed envelopes), describing any steps to conceal the sequence until interventions are assigned	10-11
Allocation: implementation	#16c	Who will generate the allocation sequence, who will enrol participants, and who will assign participants to interventions	10-11
Blinding (masking)	#17a	Who will be blinded after assignment to interventions (eg, trial participants, care providers, outcome assessors, data analysts), and how	10-11
Blinding (masking): emergency unblinding	#17b	If blinded, circumstances under which unblinding is permissible, and procedure for revealing a participant's allocated intervention during the trial	10-11
**Methods: Data collection, management, and analysis**			
Data collection plan	#18a	Plans for assessment and collection of outcome, baseline, and other trial data, including any related processes to promote data quality (eg, duplicate measurements, training of assessors) and a description of study instruments (eg, questionnaires, laboratory tests) along with their reliability and validity, if known. Reference to where data collection forms can be found, if not in the protocol	19-22
Data collection plan: retention	#18b	Plans to promote participant retention and complete follow-up, including list of any outcome data to be collected for participants who discontinue or deviate from intervention protocols	19-22
Data management	#19	Plans for data entry, coding, security, and storage, including any related processes to promote data quality (eg, double data entry; range checks for data values). Reference to where details of data management procedures can be found, if not in the protocol	19-22

Statistics: outcomes	#20a	Statistical methods for analysing primary and secondary outcomes. Reference to where other details of the statistical analysis plan can be found, if not in the protocol	20-22
Statistics: additional analyses	#20b	Methods for any additional analyses (eg, subgroup and adjusted analyses)	20-22
Statistics: analysis population and missing data	#20c	Definition of analysis population relating to protocol non-adherence (eg, as randomised analysis), and any statistical methods to handle missing data (eg, multiple imputation)	20-22
**Methods: Monitoring**			
Data monitoring: formal committee	#21a	Composition of data monitoring committee (DMC); summary of its role and reporting structure; statement of whether it is independent from the sponsor and competing interests; and reference to where further details about its charter can be found, if not in the protocol. Alternatively, an explanation of why a DMC is not needed	20-22
Data monitoring: interim analysis	#21b	Description of any interim analyses and stopping guidelines, including who will have access to these interim results and make the final decision to terminate the trial	20-22
Harms	#22	Plans for collecting, assessing, reporting, and managing solicited and spontaneously reported adverse events and other unintended effects of trial interventions or trial conduct	20-22
Auditing	#23	Frequency and procedures for auditing trial conduct, if any, and whether the process will be independent from investigators and the sponsor	19-22
**Ethics and dissemination**			
Research ethics approval	#24	Plans for seeking research ethics committee / institutional review board (REC / IRB) approval	8-9,22
Protocol amendments	#25	Plans for communicating important protocol modifications (eg, changes to eligibility criteria, outcomes, analyses) to relevant parties (eg, investigators, REC / IRBs, trial participants, trial registries, journals, regulators)	8-9,22

For peer review only

Consent or assent	#26a	Who will obtain informed consent or assent from potential trial participants or authorised surrogates, and how (see Item 32)	9-10,22
Consent or assent: ancillary studies	#26b	Additional consent provisions for collection and use of participant data and biological specimens in ancillary studies, if applicable	9-10,22
Confidentiality	#27	How personal information about potential and enrolled participants will be collected, shared, and maintained in order to protect confidentiality before, during, and after the trial	19-22
Declaration of interests	#28	Financial and other competing interests for principal investigators for the overall trial and each study site	26-27
Data access	#29	Statement of who will have access to the final trial dataset, and disclosure of contractual agreements that limit such access for investigators	22
Ancillary and post trial care	#30	Provisions, if any, for ancillary and post-trial care, and for compensation to those who suffer harm from trial participation	19-20,22
Dissemination policy: trial results	#31a	Plans for investigators and sponsor to communicate trial results to participants, healthcare professionals, the public, and other relevant groups (eg, via publication, reporting in results databases, or other data sharing arrangements), including any publication restrictions	22
Dissemination policy: authorship	#31b	Authorship eligibility guidelines and any intended use of professional writers	22,26-27
Dissemination policy: reproducible research	#31c	Plans, if any, for granting public access to the full protocol, participant-level dataset, and statistical code	8-9, 22
**Appendices**			
Informed consent materials	#32	Model consent form and other related documentation given to participants and authorised surrogates	Appendix
Biological specimens	#33	Plans for collection, laboratory evaluation, and storage of biological specimens for genetic or molecular analysis in the current trial and for future use in ancillary studies, if applicable	14,18-19

None The SPIRIT checklist is distributed under the terms of the Creative Commons Attribution License CC-BY-ND 3.0. This checklist can be completed online using https://www.goodreports.org/, a tool made by the EQUATOR Network in collaboration with Penelope.ai

For peer review only
